# Instant Writing of Conductive Interface on MOF Single Crystal by Ultrafast Laser

**DOI:** 10.1002/advs.202500711

**Published:** 2025-06-05

**Authors:** Dongsheng Huang, Shuailong Guo, Peng Chen, Yanan Liu, Zhenhua Wang, Ye Ding, Hao Li, Huijun Wu, Zhiyuan Ma, Haoqing Jiang, Lijun Yang, Hongxing Xu

**Affiliations:** ^1^ Zhenzhou Research Institute Harbin Institute of Technology Zhengzhou 450000 China; ^2^ Institute of Laser Manufacturing Henan Academy of Sciences Zhengzhou 450000 China; ^3^ School of Mechatronics Engineering Harbin Institute of Technology Harbin 150001 China; ^4^ School of Materials Science and Engineering Harbin Institute of Technology Harbin 150001 China

**Keywords:** electronics, interface, laser metallurgy, MOF single crystal, sensor

## Abstract

Single crystals with excellent properties have been widely used in electronics industries due to their homogeneous and consistent structures. Metal‐organic frameworks (MOFs), as a class of crystalline materials that can be synthetically tuned for functionality, are expected to be a favorable candidate for novel electronic devices. However, there is still a lack of methods to efficiently fabricate conductive patterns at the single‐crystal scale. Here, laser instant writing of in situ continuous conductive interface on MOF single crystals is reported, enabling the patterning and continuous fabrication of conductive interface at the single‐crystal scale. Carbon‐wrapped Cu nanoparticles (Cu@C NPs) conductive interface is instantly written using a 1030 nm picosecond ultrafast laser on large HKUST‐1 single crystals. It is found that different thermal accumulations can affect the conductivity of Cu@C and transformation of matter phase from Cu NPs to Cu_2_O on single crystals is observed as the ablation of carbonaceous materials. As a validation, single‐crystal sensor with interdigitated electrodes (IDEs) constructed by laser interface technique shows a wide response range of 5%–90% RH and a fast response time of 2 s toward humidity sensing. This method sheds new light on the construction of functional interface on single MOF crystal, providing a novel strategy for MOF‐based electronics.

## Graphical abstract

Current device fabrication strategies lack capacity for tailoring ideal electrical interface in individual MOF single crystals. An ultrafast laser approach is developed that in situ constructs stable conductive interface on MOF single crystal scale while maintaining its crystalline integrity. This strategy enables one‐step transformation of MOF single crystals into both fabrication platforms and functional devices.

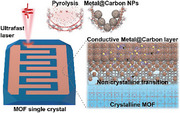

## 1 Introduction

The intrinsic advantages of single‐crystal materials have led them to achieve a wide range of applications in industrial fields. As a representative porous material, Metal‐Organic Frameworks (MOFs) single crystals provide a wealth of possibilities at the application level due to their unique physicochemical properties. An intact MOF single crystal naturally avoid the grain boundaries, defects, and impurities, meanwhile, its original monolithic structure gives it high bulk performance in micro‐optical and electronic devices.^[^ ^1^, ^2^, ^3^, ^4^, ^5^, ^6^, ^7^ ^]^ These properties make single‐crystal MOFs highly promising for integrated electrical devices. Fabrication of patterned and conductive microstructures for them is playing a key role in wide applications such as microelectronics,^[^ ^8^, ^9^, ^10^, ^11^, ^12^, ^13^ ^]^ information storage,^[^ ^3^, ^5^, ^6^, ^14^, ^15^ ^]^ and sensing.^[^ ^16^, ^17^, ^18^ ^]^ However, current generic subtractive/formative micro/nanofabrication strategies including laser ablation,^[^ ^13^, ^18^, ^19^, ^20^, ^21^, ^22^ ^]^ photolithography and electron beam etching,^[^ ^12^, ^23^, ^24^, ^25^ ^]^ are mainly targeted at MOF powders or films,^[^ ^26^, ^27^ ^]^ which leave the processing of single‐crystal MOFs relatively unexplored due to their processing and electrical integration challenges.

During the fabrication of MOF single‐crystal devices, the good crystal‐electrode interface is a necessary prerequisite for the signal response of the device.^[^ ^28^ ^]^ Conductive structures fabricated by physical bonding of metal electrodes to single crystal interface^[^ ^29^, ^30^, ^31^, ^32^, ^33^ ^]^ or by electron beam deposition^[^ ^34^, ^35^, ^36^, ^37^, ^38^, ^39^ ^]^ have been reported in 2D and 3D MOF single‐crystal device applications, which their interfacial stability and process complexity remain technical issues to be considered. Meanwhile, the potential for constructing in‐situ conductive structures directly from single crystals themselves has been overlooked, owing to the lack of feasible fabrication approaches. Several researchers have made progress in the preparation of metallic structures through external natural growth transfer printing^[^ ^7^ ^]^ and internal photocatalytic reduction.^[^ ^14^ ^]^ However, the randomness of the growth location can lead to the integrity and location of the embedded metal structure not being reproducible. For photocatalytic reduction inside single crystal, only intermittent metal features can be produced, which cannot effectively form a conductive continuous metal pattern.

Focused laser acts as an effective heat source which has been demonstrated with significant advantages in inducing derived multifunctional nano‐materials from MOFs.^[^ ^40^, ^41^ ^]^ This unique capability provides possibilities for novel derivative micro/nanoscale processing on MOF single crystals. Herein, we report the construction of conductive interface at the MOF single‐crystal scale using a 1030 nm picosecond pulsed laser. This is achieved by ultrafast laser instant writing on the surface of MOF, where coordinated metal atoms are reduced to carbon‐wrapped uniformly distributed metal nanoparticles (NPs) during the pyrolysis of organic ligands. In this way, conductive patterning interface on single MOF crystals can be generated instantly. To verify the feasibility of ultrafast laser method, large HKUST‐1 single crystals^[^ ^42^ ^]^ with high absorption at 1030 nm are chosen.^[^ ^13^ ^]^ Laser‐induced pyrolysis reduces Cu ions to metallic Cu encapsulated within carbonaceous materials while preserving the unprocessed areas’ crystalline structure. The effective ablation heat of irradiation from the surface to the interior gradually decreases, leading to the natural formation of a continuous transition interface consisted of carbon skeleton and non‐crystalline MOF structures with the aggregation of Cu NPs. Instant laser writing is a simple one‐step process whose programmability ensures the micropatterning of the conductive interface. By modulating the scanning speed and laser power, we hypothesize that the denaturation of the carbonaceous material caused by thermal accumulation affects the conductivity of the carbon‐wrapped Cu (Cu@C) layer. The ablation of the carbonaceous material leads to the transformation from Cu to Cu_2_O NPs, which is expected to combine single crystals with semiconductor materials. Furthermore, we validated this device fabrication strategy through humidity sensing tests after creating interdigitated electrodes (IDEs) on HKUST‐1 via ultrafast laser writing. The fabricated HKUST‐1 single crystal sensor shows a wide detection range of 5%–90% RH, with the fastest response time of only 2 s. This work establishes a foundation for developing a novel MOF single crystal devices fabrication strategy and paves the way for further advancements in the field.

## 2 Results and Discussions

### 2.1 Fabrication of Laser‐Induced Cu@C on HKUST‐1 Single Crystal

Large crystals of HKUST‐1 are synthesized according to the previous report,^[^ ^42^ ^]^ and the diffraction profiles match well with HKUST‐1 by powder X‐ray diffraction (PXRD) (Figure S1, Supporting Information). HKUST‐1 demonstrate an absorptivity of up to 70% at 1030 nm due to the secondary building units (SBUs) consisting of copper ions and BTC ligand.^[^ ^13^ ^]^ The short time‐domain property of ultrafast laser allows the diffusion of heat confined to the spot effect,^[^ ^43^ ^]^ thus providing precise reduction of energy during induced processing on HKUST‐1 single crystals.

**Figure 1:**
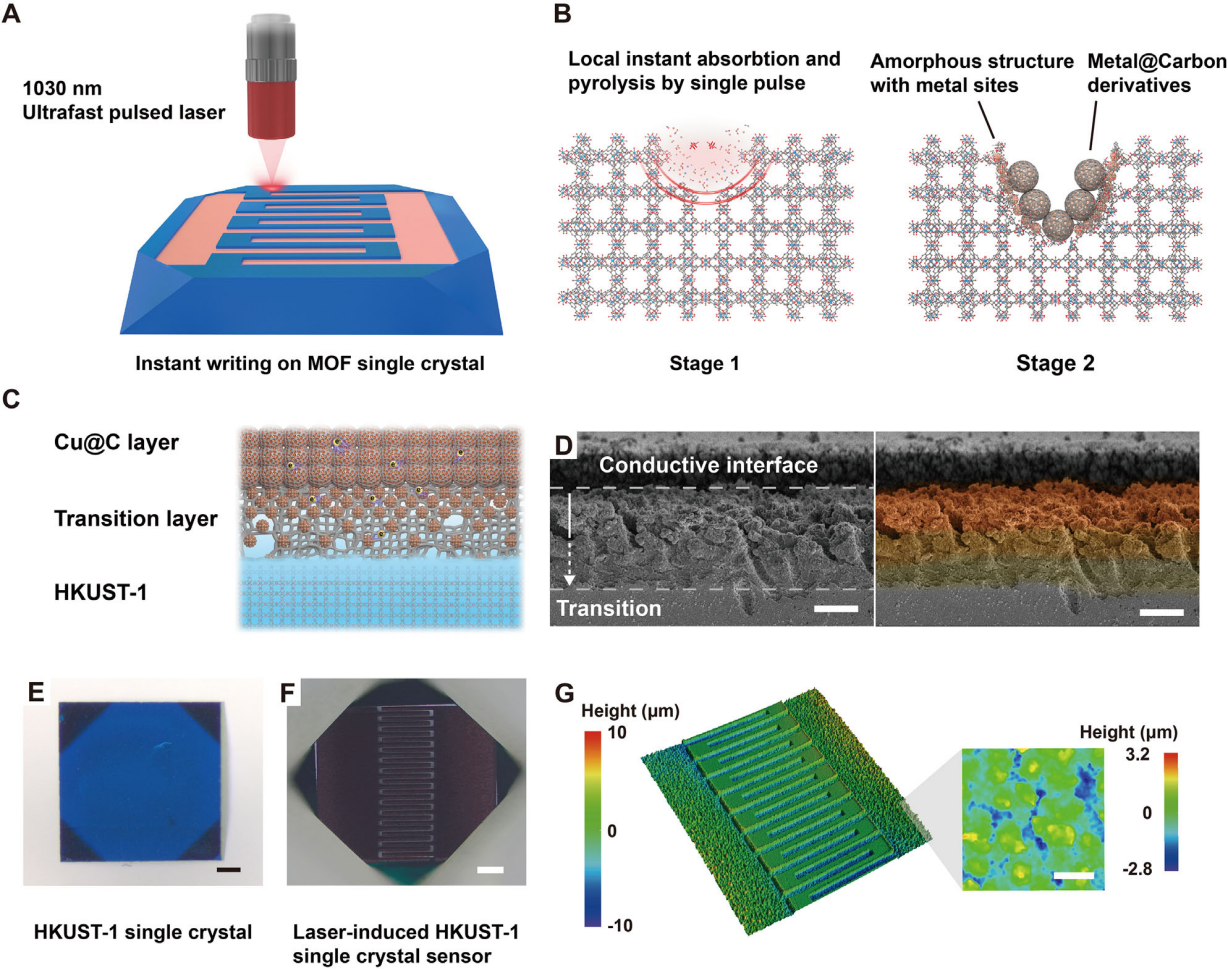
A) Schematic illustrations of ultrafast pulsed laser‐induced processing of HKUST‐1 single crystal. The objective lens focuses a small spot onto the surface of a single crystal. B) Mechanism for generating Metal@Carbon derivatives and amorphous layer by ultrafast pulsed laser. The coordinated metals in SBUs absorb most of the photons, rendering the pyrolysis and thermal shocks (presented in red) in localized regions. C) Schematic diagram of the conductive interface onto the crystalline structure. The Cu NPs are presented in yellow. D) Side view SEM image of the conductive interface. The degree of color represents a gradual decrease for the content of Cu NPs. E,F) Optical microscopy image of HKUST‐1 single crystal. G) 3D profile image of laser‐induced IDEs on single crystal. Scale bars of 5 µm for (D), 100 µm for (E,F), and 4 µm for (G).

As can be seen from **Figure** **1**A,B, the irradiated material serves as a precursor for derivation when the laser is focused on the surface of HKUST‐1 single crystal. Pyrolysis of organic ligands caused by localized transient heating generates a reducing atmosphere (composed by H_2_, CO, CH_4_, and other gases) that reduces coordinated metal ions to metallic NPs (Cu^2+^ to Cu^0^).^[^ ^13^ ^]^ These NPs aggregate and are encapsulated by thermally decomposed carbonaceous materials to form Cu@C nano‐structures. Amorphization of the crystal structure occurs at the laser ablation edge due to the dissipation of heat conduction. This process results in the formation of an incompletely induced amorphous transition layer with aggregated metal sites onto the intact crystal structure. When large regional processing is performed, the conductive interface consisting of Cu@C and amorphous layer with high‐density metal sites can be produced on the crystalline structure (Figure 1C). The surface layer of the interface is Cu@C produced by completely inducing and the density of Cu NPs scattered in carbon skeleton and non‐crystalline structures gradually decreases as the color shown in Figure 1D. Scanning electron microscopy (SEM), energy‐dispersive X‐ray spectroscopy (EDS), and optical microscopy images demonstrate the amorphous layer as a substrate for supporting aggregated Cu@C NPs and the continuous derivative transition onto crystalline interface (Figures S2 and S3, Supporting Information). Previous studies utilizing laser ablation of MOF powders resulted in complete destruction of the original crystalline frameworks, failing to find or exploit the potential synergistic effects between the derived materials and pristine MOF single crystals. This stands in fundamental contrast to our ultrafast laser direct writing approach, which maintains the structural integrity of unirradiated regions, preserving the intrinsic crystalline properties.

HKUST‐1 single crystals with flat surfaces are selected as samples to be processed (Figure 1E). After patterned processing on single crystals, precise IDEs with uniform intervals are obtained on the flat crystal surface (Figure 1F,G) while the remaining intact structure of the single crystal will preserve its intrinsic properties. Compared with other fabrication strategies in Table S1 (Supporting Information), the laser interface technique readily enables the single crystal itself to become a manufacturing platform without other procedures, providing new technological possibilities for the fabrication and integration of MOF single‐crystal microelectronic devices.

### 2.2 Analytical Characterization of MOF‐Derived Cu@C and Cu_2_O NPs Obtained by Different Processes

#### 2.2.1 MOF‐Derived Cu@C Produced by Single Scan Processing

**Figure 2:**
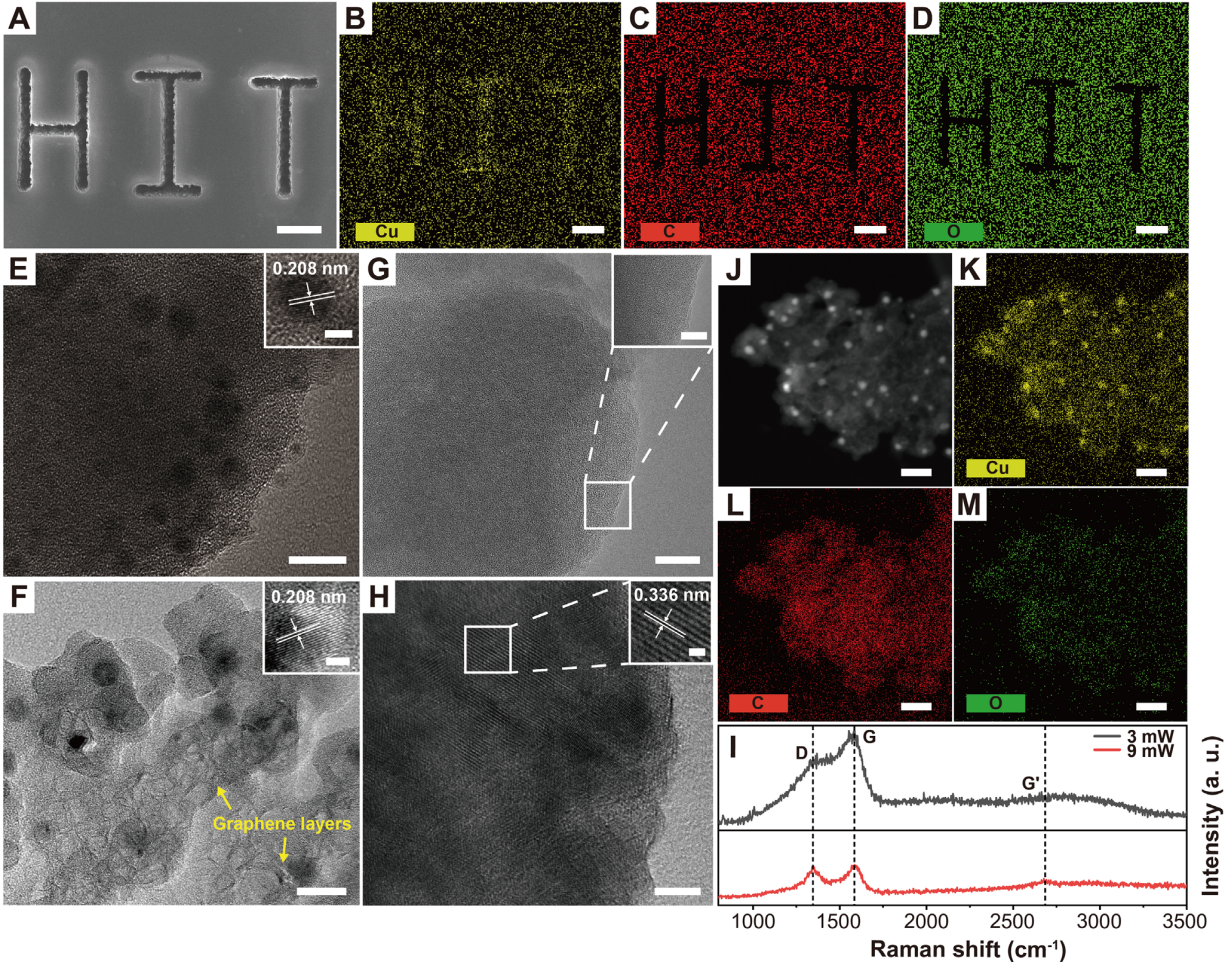
Characterization of Cu@C produced by laser single scan processing. A–D) SEM and EDS images after laser‐induced processing on HKUST‐1 single crystal. HR‐TEM images show Cu@C NPs produced at E) 3 mW and F) 9 mW. HR‐TEM images of the derived carbon materials demonstrate the amorphous carbon is produced at G) 3 mW, while the carbonaceous material at H) 9 mW have a better degree of graphitization. I) Raman spectra of 3 and 9 mW processing. J–M) EDS‐TEM images of Cu@C NPs produced by single scan processing at 9 mW. Scale bars of 15 µm for (A–D), 10 nm for (E), 20 nm for (F,G), 5 nm for (H), 2 nm for the inset of (E,F,H), 5 nm for the inset of (G), and 50 nm for (J–M).

Optical microscopic and SEM images demonstrate the feasibility of ultrafast laser processing on HKUST‐1 single crystal (Figure S4, Supporting Information). SEM‐EDS images (**Figure** **2**A–D) display the removal of C and O after 3 mW irradiation, while the Cu content increases compared to the intact area. EDS line scan confirms an elevated Cu content and a significant reduction in C and O after irradiation. The edges of the irradiated area also show an increase in the Cu content, presenting a thermal reduction of the metal without destroying the surface macrostructure (Figure S5, Supporting Information). The relative Cu content following 9 mW processing is significantly higher compared to 3 mW, showing that within a specific thermal range, increased heat promotes a greater degree of Cu reduction. High‐resolution TEM (HR‐TEM) analysis in Figure 2E,F indicates the generation of Cu@C NPs by single scan processing. Distinct lattice fringes with interplanar distances of 0.208 nm in the (111) crystal plane of Cu are observed for samples processed at 3 mW (Figure 2E) and 9 mW (Figure 2F), respectively. After laser irradiation, different laser powers result in different degrees of graphitization of the derived carbon materials. HR‐TEM at 3 mW (Figure 2E,G) presents a disordered carbon structure, whereas HR‐TEM at 9 mW (Figure 2F,H) clearly reveals multilayered graphene structures and graphite lattice fringes with interplanar distances of 0.336 nm in the (002) crystal plane.^[^ ^13^ ^]^ Figure 2I shows the Raman spectra of the derived carbon materials at 3 and 9 mW processing power. The G and D peaks presented in high sp^2^‐carbon content amorphous carbon are observed.^[^ ^44^ ^]^ The narrowing and separation of the two peaks suggest that higher power (9 mW, ID/IG = 0.96) induces greater graphitization compared to lower power (3 mW, ID/IG = 1.07). This observation is consistent with prior reports that different laser powers affect the degree of graphitization of carbonaceous materials produced after pyrolysis of organic ligands.^[^ ^19^ ^]^ Additionally, EDS‐TEM images (Figure 2J–M) demonstrate uniform distributions of Cu, C, and O, confirming the encapsulation of Cu NPs by carbonaceous materials.

#### 2.2.2 MOF‐Derived Cu@C and Cu_2_O Layer Produced by Scanning Processing Line‐by‐Line

**Figure 3:**
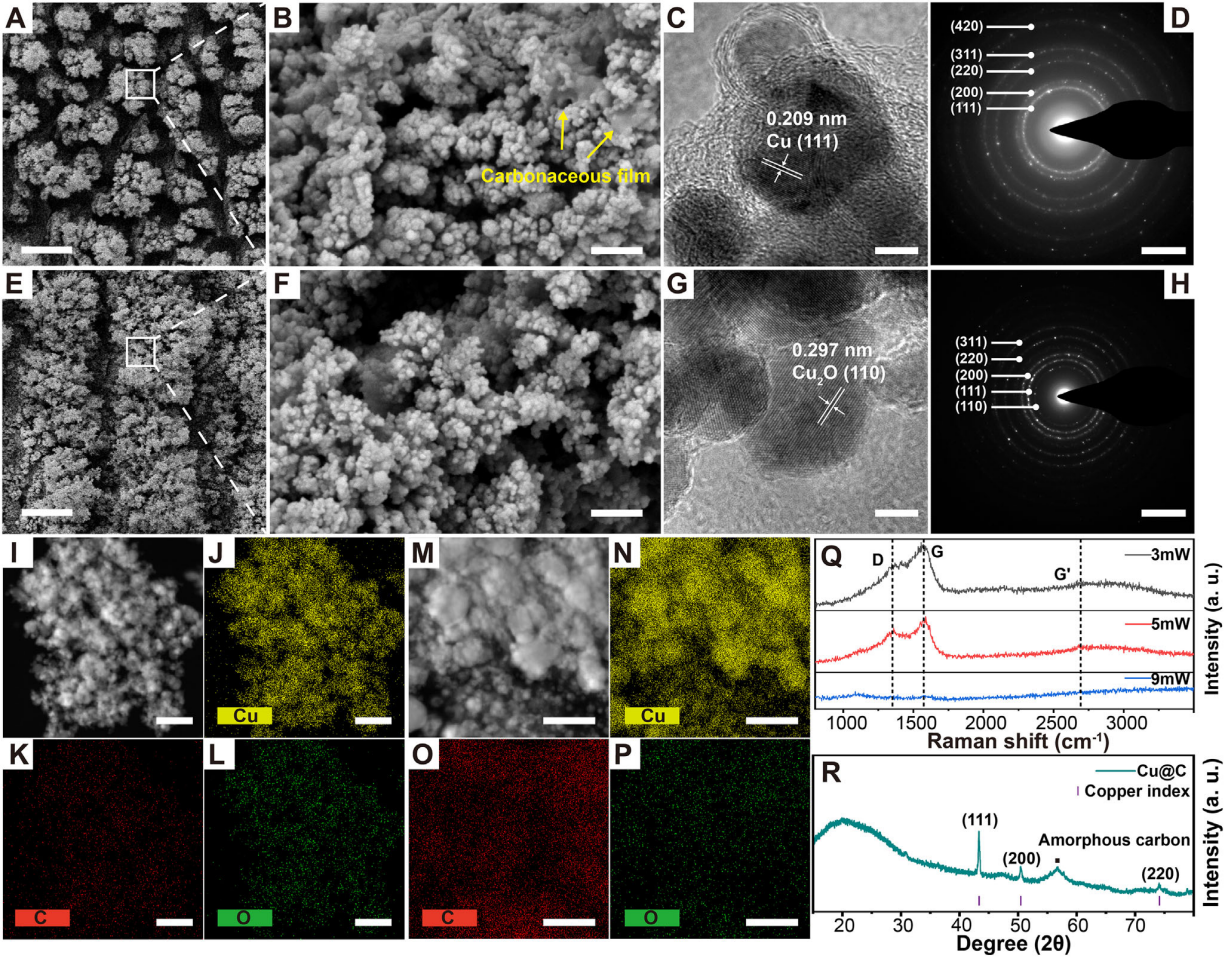
Characterization of derivatives produced by laser processing line‐by‐line. Low and high (80K X) magnification SEM images of A,B) 3 mW and E,F) 9 mW. HR‐TEM images of C) 3 mW and G) 9 mW. SAED patterns match well with metallic Cu at D) 3 mW and Cu_2_O at H) 9 mW. EDS‐TEM images of MOF metal derivatives after I–L) 9 mW and M–P) 3 mW. Q) Comparison of Raman spectra after different power processing. R) Micro‐focal XRD spectra at 5 mW. Scale bars of 4 µm for (A,E), 200 nm for (B,F), 5 nm for (C,G), 5 nm^−1^ for (D,H), and 100 nm for (I–P).

Patterned preparation of planar electrical interface on the surface of MOF single crystals requires regional laser processing (line‐by‐line) to form functional electrical regions. Areas irradiated with different laser powers at equal intervals exhibited reddish‐brown (3 mW) and yellowish‐green (9 mW) colors under the optical microscope, respectively, as shown in Figure S6 (Supporting Information). Processing interval was appropriately performed at 1 µm as described in Figure S7 (Supporting Information). **Figure** **3**A–D and Figure 3E–H display the microscopic characterization after irradiating with 3 and 9 mW laser power, respectively. The SEM image in Figure 3A shows small petal‐like protruding structures, while the structures in Figure 3E become larger. The accumulation of transient high heat followed by rapid cooling explains the sputtering and reaggregation of the derived material, resulting in these unique structures. Aggregated Cu@C NPs and some carbonaceous film can be observed from the magnification image (Figure 3B). Further, the HR‐TEM image and selected area electron diffraction (SAED) pattern clearly demonstrate the lattice fringes (Figure 3C) and diffraction circles corresponding to the 111, 200, 220, 311, and 420 facets of metallic Cu (Figure 3D). On the other hand, the same TEM analysis demonstrates the lattice fringes and the 110, 111, 200, 220, and 311 facets of Cu_2_O NPs produced by higher heat accumulation, as shown in Figure 3G,H. Power conditions tests between these two phases of matter were also applied. As the power increases, the transition from Cu to Cu₂O begins to occur. At a power of 5 mW, a pure phase of Cu is still observed. However, at 8 mW, the selected area electron diffraction (SAED) pattern indicates the emergence of a mixed phase, characterized by the presence of a small amount of Cu₂O alongside a predominant Cu phase (Figure S8, Supporting Information).

The region after 9 mW irradiation has almost no visible carbonaceous material encapsulating Cu_2_O NPs (Figure 3F,G; Figure S9, Supporting Information). It is also evident from the EDS‐TEM images (Figure 3I‐L) that the carbon signal in the region is weak whereas the signal of carbon encapsulation on the 3 mW (Figure 3M‐‐P) region is more pronounced. The disappearance of D and G bands in the Raman spectrum proves the ablation removal of carbonaceous materials after 9 mW irradiation (Figure 3Q). These results suggest that the conversion of Cu@C to Cu_2_O NPs is due to the removal of the carbonaceous material under the higher heat ablation. At the same time, Cu NPs undergo thermal oxidation to produce Cu_2_O NPs (Cu^0^ to Cu^+^). Carbon‐wrapped Cu and unwrapped Cu_2_O NPs were found in the derivatives, illustrating the protection by wrapped carbonaceous materials (Figure S10, Supporting Information). Moreover, the expansion of ablation size and an uneven distribution of color and structure is observed on the irradiated area at 9 mW from the optical microscope image, reflecting the presence of a mixture of Cu_2_O and Cu@C (Figure S11, Supporting Information). Figure S12 (Supporting Information) shows the distribution of the remaining Cu@C mixed with Cu_2_O after thermal oxidation at 9 mW compared to other lower power (acid treatment conditions). The uniformity of Cu NPs in the surface region of the single crystal after 5 mW laser irradiation was confirmed by a micro‐focal spot X‐ray diffractometer (XRD) with a spot diameter of 300 µm. Accompanied by the diffraction peaks of amorphous carbon, the three fingerprint peaks located at 2θ = 43.3, 50.4, and 74.1° in the micro‐focal XRD pattern correspond well to the 111, 200, and 220 facets of metallic Cu (Figure 3R).

### 2.3 Analysis of Different Processes on Electrical Properties

**Figure 4:**
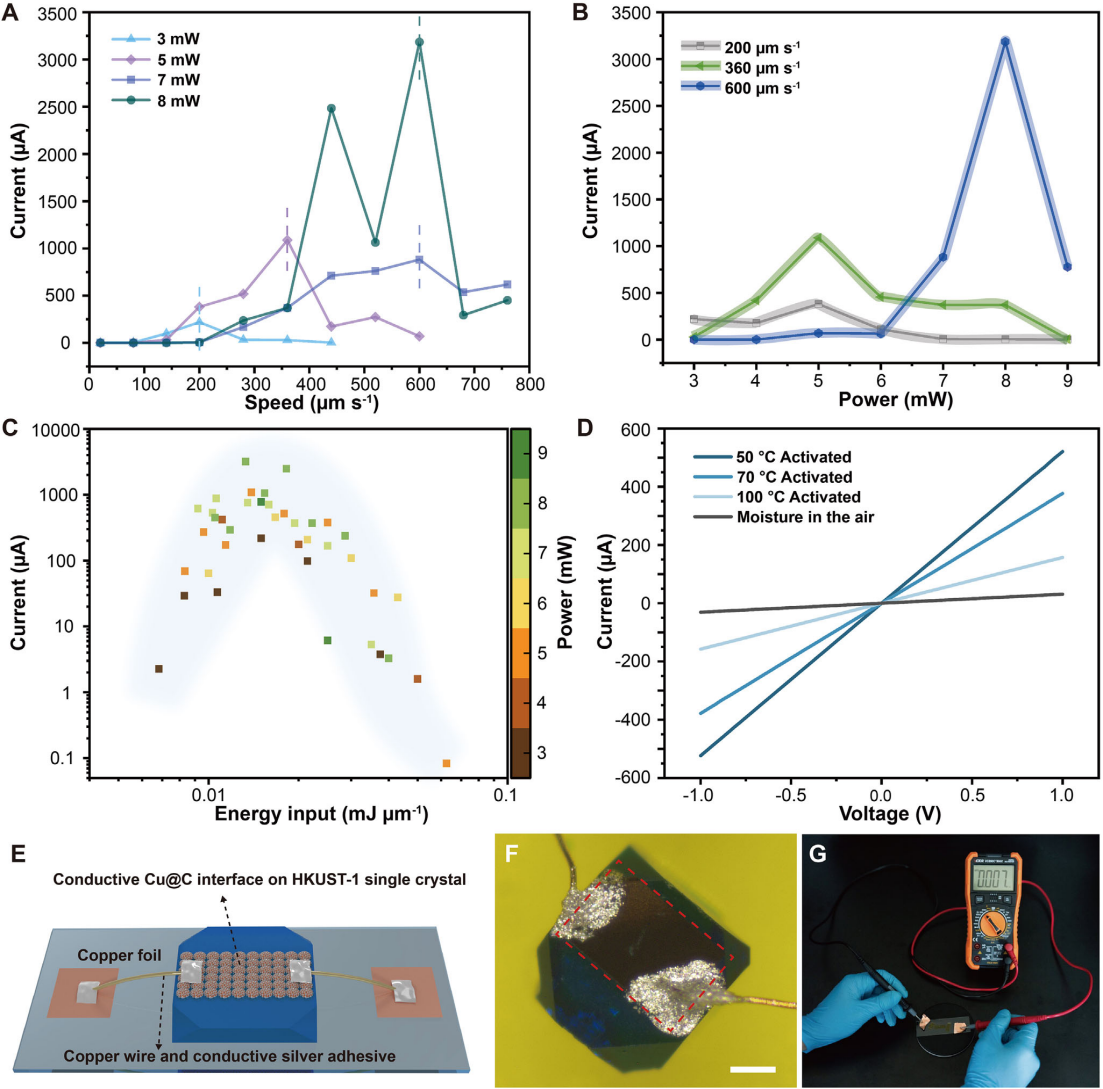
Electrical properties measurements and analysis of conductive interface prepared by ultrafast pulsed laser on HKUST‐1 single crystal. A) Changes of current values in the conducting layer with the scanning speed at different powers. B) The current values vary as a function of the laser power at different speeds. C) Distribution of current magnitude as single crystals receive different energy inputs. D) Electrical properties of HKUST‐1 single crystals at different activation temperatures. E) Schematic illustration of Cu@C bonding on single crystal. F) Optical microscope image after connecting the wires. G) Resistance tests of single crystal connected to external circuit after bonding. Scale bar of 200 µm for (F).

Metal@Carbon conductive interface produced on intact MOF single crystal via ultrafast laser‐induced processing holds significant promise for electronic device applications. To explore and analyze this process, a two‐probe *I–V* measurement (with a voltage of 1 V) was performed on Cu@C layers processed with different laser powers. The ratio of power to scanning speed was defined as the energy input per unit length of the laser process to reflect the degree of heat accumulation. Different power conditions reflect that the current values decrease at both high and low scanning speeds (**Figure** **4**A). Maximum current is achieved at moderate scanning speeds (indicated by dashed lines). Current measurements in Figure 4B show that excessive or insufficient thermal accumulation reduces conductivity. Maximum currents were obtained at 5 mW for low and medium speeds (0.38 mA at 200 µm s⁻¹ and 1.1 mA at 360 µm s⁻¹) and 8 mW for higher speed (3.2 mA at 600 µm s⁻¹). Figure 4C clearly presents the distribution of heat accumulation versus current values, with high current values mainly distributed at 0.01–0.018 mJ µm^−1^. Low energy inputs are insufficient to facilitate the formation of a continuous and complete Cu@C layer, resulting in reduced conductivity due to incomplete coverage and inadequate connectivity of the conductive pathways. Whereas other degrees of thermal accumulation affect the conductivity of the wrapped carbonaceous material and in the case of higher heat accumulation, even insulation will occur. TEM and optical microscopy analysis of the insulating case show that carbonaceous material and Cu NPs are still present after high energy inputs, but some metallic Cu is thermally oxidized to Cu_2_O due to carbon ablation (Figure S13, Supporting Information). The partial presence of Cu_2_O does not result in insulation. Comparing the effect of power on the degree of graphitization in Figure 2E–H, the TEM images of the carbon material with Cu@C produced by 10 times larger heat accumulation at the same power show only amorphous morphology (Figure S14, Supporting Information). We hypothesize that the insulating situation before the complete transformation of Cu into Cu_2_O is mainly due to the higher energy input, causing an increase in disorder and a decrease in the density of conducting sites during carbonaceous material formation,^[^ ^45^ ^]^ which ultimately leads to the creation of a Metal@Insulating Carbon (Cu@IC) material. Figure S15 (Supporting Information) shows the gradual conversion of the main metal derivatives from Cu@IC to Cu_2_O with increasing heat accumulation under insulating conditions. The diffuse broadband in the Raman spectra reflects the structural variability of the carbonaceous material in the insulating and conducting situations (Figure S16, Supporting Information), which is consistent with our hypothesis.

To investigate the effect of single crystal synthesis conditions on electrical properties, measurements at different activation temperatures and different solution immersions were carried out. Figure 4D shows a decrease in current with the increase of activated temperatures due to the decrease of coordinated water molecules. Additionally, the HKUST‐1 single crystal damped in the air for a long time is susceptible to fragmentation of the conductive interface and significant current reduction after laser processing. However, filling the sample with water prior to processing significantly increases the conductivity of conductive interface, attributed to the penetration of the water layer under the intact conductive interface through the porous network structure (Figure S17, Supporting Information). This also suggests that conductivity occurs not only across the interface, but that the native structure at the bottom of the interface is also involved in current conduction. We used conductive silver paste to bond micro‐sized copper wires on the conductive surface of the single crystal to achieve loop conduction (Figure 4E–G). The degree of oxidation can be directly assessed through resistance measurements, as changes in resistance values correlate with the extent of oxidation. The test resistance was stabilized at ≈4 times the initial value with a gradual decrease in the average oxidation rate, indicating the electrical properties of the conductive interface are stable (Figure S18, Supporting Information).

### 2.4 Laser‐Induced HKUST‐1 Single‐Crystal Resistive Sensor

**Figure 5:**
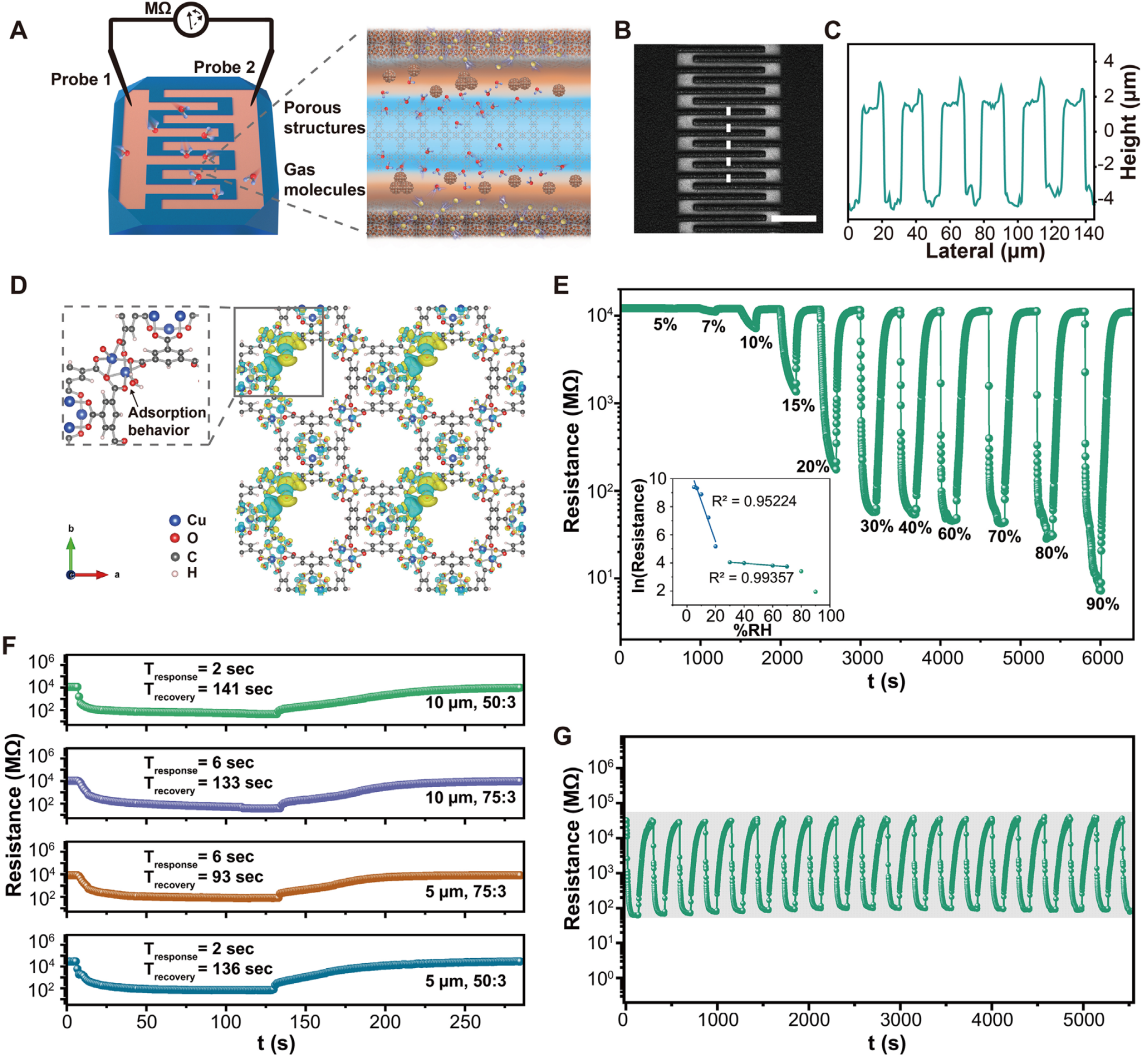
Sensing performance and analysis of HKUST‐1 single crystal humidity sensor prepared by ultrafast laser. A) Schematic diagrams of HKUST‐1 single crystal sensor. B,C) Grey‐scale image and uniform height profile of laser‐induced IDEs on single crystal measured by 3D confocal laser microscopy. D) DFT simulation of charge density difference distribution at Cu adsorption site in single crystal pores. E) Continuous response and recovery curves of the single‐crystal HKUST‐1 sensor to different relative humidities (RH). F) Comparison of the response and recovery times of different electrode structures toward 70% RH. G) Repeatability tests in cyclic humidity environments. Scale bar of 100 µm for (B).

Combined with the intrinsic adsorption advantages of MOF materials, a novel single‐crystal resistive sensor can be prepared by laser‐induced conductive interface on MOF single crystal. When the HKUST‐1 single crystal is placed in a dynamically gassed humidity environment, it can spontaneously adsorb the surrounding gas molecules due to the porous properties and the molecules concentration difference. After laser direct writing of IDEs on single crystal, a conductive patterning interface is formed as the electrode fingers for electrical signal transmission. The crystalline MOF and the amorphous interfacial transition layer (with abundant metal sites) between electrode fingers can serve as the primary response materials which may have a strong adsorption effect on water molecules and lead a fast electrical response to humidity (**Figure** **5**A). Figure S19 (Supporting Information) shows the diagram of the electrodes feature and the optical microscopy images on crystal. The programmability of ultrafast laser processing enables the fabrication of diverse electrical structures. The 3D profile of the patterned Cu@C IDEs and their periodic uniform height profile are shown in Figure 5B,C, which demonstrates the efficiency and feasibility of the laser processing method. Density functional theory (DFT) calculations were carried out to get an adsorption energy of 0.2928 and 0.1239 eV for the Cu sites and the vicinity of benzene ring, confirming the adsorption of water molecules inside the pores in the humidity atmosphere (Figure 5D; Supporting Note). The single crystals can be regarded as a gas reservoir to gradually fill with water molecules from the surroundings to the bottom of electrode interface, which can stabilize the response value when the adsorption reaches a dynamic equilibrium. Physical adsorption process based on van der Waals forces and hydrogen bonds is more likely to be formed between the water molecules and the adsorption sites.^[^ ^46^ ^]^ When dry air is purged into the pores, aggregated water molecules under adsorption will be squeezed out and carried away through the interaction force generated by the influx of dry air, enabling full sensor recovery.

Before testing, the samples are exposed to dry air with relative humidity below 5% for a specific duration to stabilize the resistance values. Continuous response/recovery test over a wide range of relative humidity (from 5% RH to 90% RH) demonstrates the excellent response and stability of the laser‐induced HKUST‐1 single crystal sensor (Figure 5E; Figure S20, Supporting Information). Table S2 (Supporting Information) demonstrates the advantages of the laser‐induced single‐crystal humidity sensor in terms of both performance and miniaturization compared to different HKUST‐1 humidity sensors. A faster response (30%–90% RH, a highly practical detection range) and a large ratio of response resistance (90%RH, 10^3^) is presented. The bulk single crystal can function as a platform to stably hold the conductive structures and also has the smaller size. Meanwhile, compared to the sensors where the entire HKUST‐1 film is deposited on the IDEs, laser‐induced single crystal sensor requires only micron active material between these fingers, avoiding the susceptibility to damage caused by excessive exposure. The response resistance and corresponding response‐humidity curves are presented in Figure S21 (Supporting Information). At low humidity, water molecules inside the pores dissociate into H^+^ and OH^−^ mainly through the action of copper and oxygen sites, enabling charge transport,^[^ ^46^ ^]^ showing a high response resistance. In this case, the increase in humidity significantly contributes to the decrease in response resistance (from 11990 to 177 MΩ, corresponding to 5% RH to 20% RH). After increasing the humidity, the pores can quickly adsorb enough water molecules for carrier transport which mainly rely on hydronium ions (H_3_O^+^) proton conduction via the Grotthuss chain reaction between the water molecules.^[^ ^47^, ^48^ ^]^ In this case, the response resistances in the high humidity range are maintained stably below 100 MΩ and only slightly decrease with increasing humidity, revealing that this humidity range is able to temporarily saturate the water molecule response (from 57 to 42 MΩ, corresponding to 30% RH to 70% RH). When the humidity reaches 90% RH, the higher concentration of water molecules in the pores form a more continuous conduction network, causing an abrupt change in the response resistance at high humidity (up to 7 MΩ). The ln(responses resistance) at low humidity (< 20% RH) exhibits a strong linear variation, whereas the responses at higher humidity (30%–70% RH) change gently but remain linear up to 0.99 (inset of Figure 5E). Response/recovery curves for different interdigitated intervals and aspect ratios at 70% RH are compared in Figure 5F. Aspect ratio of 50:3 shows a fast response time (only 2 s) whereas a longer response time (6 s) is obtained at 75:3 for more water molecules absorbed. The conduction of electrical signals over long sensitive areas requires a longer adsorption process of water molecules and is therefore more susceptible to sudden changes in humidity. As a result, the aspect ratio of 75:3 makes it easier to recover the resistance value. In particular, the recovery performance is more pronounced at smaller interval (only 93 s compared to others). On the other hand, cyclic repeatability tests in 70% RH atmosphere are performed to confirm the good stability of the laser‐induced single‐crystal sensor (Figure 5G).

## 3 Conclusion

We report the instant writing of conductive micropattern interface on HKUST‐1 single crystal by an ultrafast pulsed laser. The short time‐domain nature of the ultrafast laser enables precise reduction processes while preserving the structural integrity of the single‐crystal. Compared with reported MOF single crystal fabrication strategies, direct laser writing enables the one‐step in‐situ fabrication of reproducible, high‐precision, and continuous electrical interfaces. Moreover, we find the conductivity of Cu@C layer can be affected by modulating the heat accumulation, and can even be converted into Cu_2_O NPs during carbon ablation in higher heat. Humidity tests on laser‐induced HKUST‐1 single‐crystal sensors show a wide response range and fast response time validating the effectiveness of laser‐induced electrical interface on MOFs. This work presents a new approach to the ever‐challenging preparation of 3D MOF single‐crystal electronic devices. Based on this method, future research will focus on 2D and composite MOF single crystals for microelectronic device fabrication to unlock more potential for MOF single crystal.

## 4 Experimental Section

### Materials

All chemicals were purchased from the supplier and used without further purification. Copper nitrate trihydrate (Cu(NO_3_)_2_·3H_2_O, 99%) and glacial acetic acid (99.5%) were purchased from MAKLIN Reagents. Benzene‐1,3,5‐tricarboxylic acid (H_3_BTC, 97%) was purchased from Leyan. N,N‐Dimethylformamide (DMF, 99.9%) was purchased from Aladdin. Ethanol absolute (99.7%) was purchased from Sinopharm Chemical Reagent. Scintillation vials of 20 mL were purchased from KIMBLE.

### Synthesis of HKUST‐1 Single Crystals

HKUST‐1 single crystals were prepared as previously reported.^[^ ^42^ ^]^ After synthesis the single crystals were solvent exchanged with fresh ethanol three times a day for three days. Vacuum activation of the single crystals was required after the solvent exchange was completed. In the experiment, it was found that the higher the activation temperature, the easier it was for the single crystals to lose the coordination water molecules and become close to a dark purple color, which could easily lead to structural damage due to the massive replenishment of coordination water when exposed to air. Since single crystals were inevitably exposed to air during laser processing and performance testing, it was necessary to test the activation conditions for single crystal samples to maintain a relatively stable state in air. Activation temperatures of 50, 60, 70, 85, and 100 °C were performed, and the single crystals activated at 100 °C showed a large number of cracks after a few hours in the air, 85 °C persisted for several days, and the activated single crystals at 70 °C and below remained intact in the air for several months without significant damage. To obtain stable HKUST‐1 single crystals while also achieving the activation effect, the single crystals treated at 70 °C were chosen as the experimental samples and kept them in an airtight container prior to use.

### Laser Processing of Metal@Carbon Derivatives

Large single crystals of suitable shape and flat surface were selected with sharp‐nosed tweezers. These samples were placed on clean slides. The metal@carbon pattern was written instantly on HKUST‐1 single crystals by irradiation with a 1030 nm picosecond pulsed laser (pulse width of 1.2 ps, effective inducing power of 1–9 mW and frequency varying with it from 50.8 to 330 K). The focal spot diameter was ≈2 µm and the laser scanning speed was 200 µm s^−1^.

### Characterization

Optical microscope images were recorded by ZEISS Axio Imager.M2m. Scanning electron microscopy images were recorded using a Thermo Scientific Helios 5 CX SEM. Single crystals were mounted on conductive carbon tape and sputtered with a certain thickness of Pt (excessive sputtering time can cause the single crystal to crack). Transmission electron microscope images were obtained with a FEI Tecnai G2 F20 TEM. For the preparation of TEM samples, a carbon support film was creatively pressed onto the surface of the induced processed sample with a slide under the microscope (the sample should not be crushed), and directly adhered parts of the derivatives through the adhesive action of the carbon film. For samples that needed to be refined, fragments of the processed area were screened for ultrasonic oscillation, which was later fished in the ethanol with the carbon support film. Raman spectra images were obtained by an Oxford WITec instrument using 532 nm laser excitation with a laser power of 1 mW. The Powder XRD pattern was recorded on a Rigaku MiniFlex600‐C diffractometer using Cu Kα radiation (λ = 1.540593 Å, 40 kV, 15 mA). Micro‐focal XRD patterns were recorded by Bruker D8 Discover diffractometer using Cu Kα radiation with a spot size of 300 µm (λ = 1.54056 Å, 50 kV, 1 mA). The 3D profile was measured by an Olympus LEXT OLS5100 3D measuring laser microscope.

### Electrical Testing

*I–V* testing of Cu@C conductive interface was performed by a KEITHLEY 2634B SourceMeter with a probe station, using two probes and a voltage of 1 V. The *I–V* curve was measured by touching the probe to the surface of the processed area under a microscope. To avoid the influence of different contact conditions and the sample itself, several contacts and tests were performed and different samples were processed under the same processing conditions, with the average of the three largest current values recorded.

### Wire Bonding

Micron‐scale copper wires bonded near the surface of single crystals using a high‐viscosity conductive silver paste under a microscope. When the conductive silver pastes dried, the resistance of the conductive Cu@C interface was stably measured. The bonding experiments reveal that a thinner adhesive contact produces a larger contact resistance, making the actual resistance value larger than that measured by direct probe contact. When the adhesive contact was thicker, the measured resistance was consistent with the probe test value.

### Humidity Sensing

The resistance response (R‐t) was measured on an AES‐4TH Probe Test Bench. Resistance was detected by touching two probes to each end of interdigitated electrodes on a single crystal using a CCD camera (testing with a voltage of 1 V and a frequence of 2 kHz). The response and recovery time were defined as the time it takes to decrease or increase from the baseline resistance to 90% of the total resistance change. The resistance value was taken as the average resistance under the corresponding humidity condition.

## Conflict of Interest

The authors declare no conflict of interest.

## Supporting information



Supporting Information

## Data Availability

The data that support the findings of this study are available from the corresponding author upon reasonable request.
