# Supplementary material for: Instant Writing of Conductive Interface on MOF Single Crystal by Ultrafast Laser
Source: Adv Sci (Weinh). 2025 Jun 5;12(30):e00711. doi: 10.1002/advs.202500711 (PMC12376616; doi:10.1002/advs.202500711)
Supplement: Supplementary file 1 — Supporting Information [file ADVS-12-e00711-s001.docx]

Instant Writing of Conductive Interface on MOF Single Crystal by Ultrafast Laser

*Dongsheng Huang^1,2,3†^, Shuailong Guo^2†^, Peng Chen^2^, Yanan Liu^1,4^, Zhenhua Wang^2^, Ye Ding^3^, Hao Li^2^, Huijun Wu^2^, Zhiyuan Ma^1,2,3^, Haoqing Jiang^2*^, Lijun Yang^1,3*^, and Hongxing Xu^2*^*

^1^Zhenzhou Research Institute, Harbin Institute of Technology, Zhengzhou 450000, China.

^2^Institute of Laser Manufacturing, Henan Academy of Sciences, Zhengzhou 450000, China.

^3^School of Mechatronics Engineering, Harbin Institute of Technology, Harbin 150001, China.

^4^School of Materials Science and Engineering, Harbin Institute of Technology, Harbin 150001, China.

† D. Huang and S. Guo contribute equally to this work.

*Corresponding author. E-mail: jianghaoqing@hnas.ac.cn

**Supporting Note: Density functional theory calculation**

First-principles computations based on density functional theory (DFT) were implemented in the Vienna Ab initio simulation package (VASP).^[1]^ The generalized gradient approximation (GGA) involving Perdew, Burke, and Ernzerhof (PBE) was used for calculating the exchange-correlation energy.^[2]^ A 400 eV cutoff energy was adopted for the plane-wave basis set in conjunction with the projector augmented wave (PAW).^[3]^ A vacuum space about 10 Å in *z*-axis was inserted in order to avoid any interlayer interactions. The adsorption energy (E_ads_) of adsorbates was calculated by the equation of E_ads_ = E_tot_ − E_A_ − E_B_, where E_tot_, E_A_, and E_B_ represent the total energy of MOF substrates and adsorbates, the isolated molecule of H_2_O, and the MOF substrates, respectively. The convergence parameters were set to 10^-5^ eV for the total energy and to 0.02 eV Å^-1^ for the residual forces on atoms. The DFT-D3 method was used for all calculations to consider the van der Waals forces. The adsorption energy for adsorption sites near the Cu and the center of the benzene ring inside the pore were calculated to be 0.2928 eV and 0.1239 eV, respectively. Initial adsorption sites are more likely to be located near the Cu.

**Supporting Figures**

**
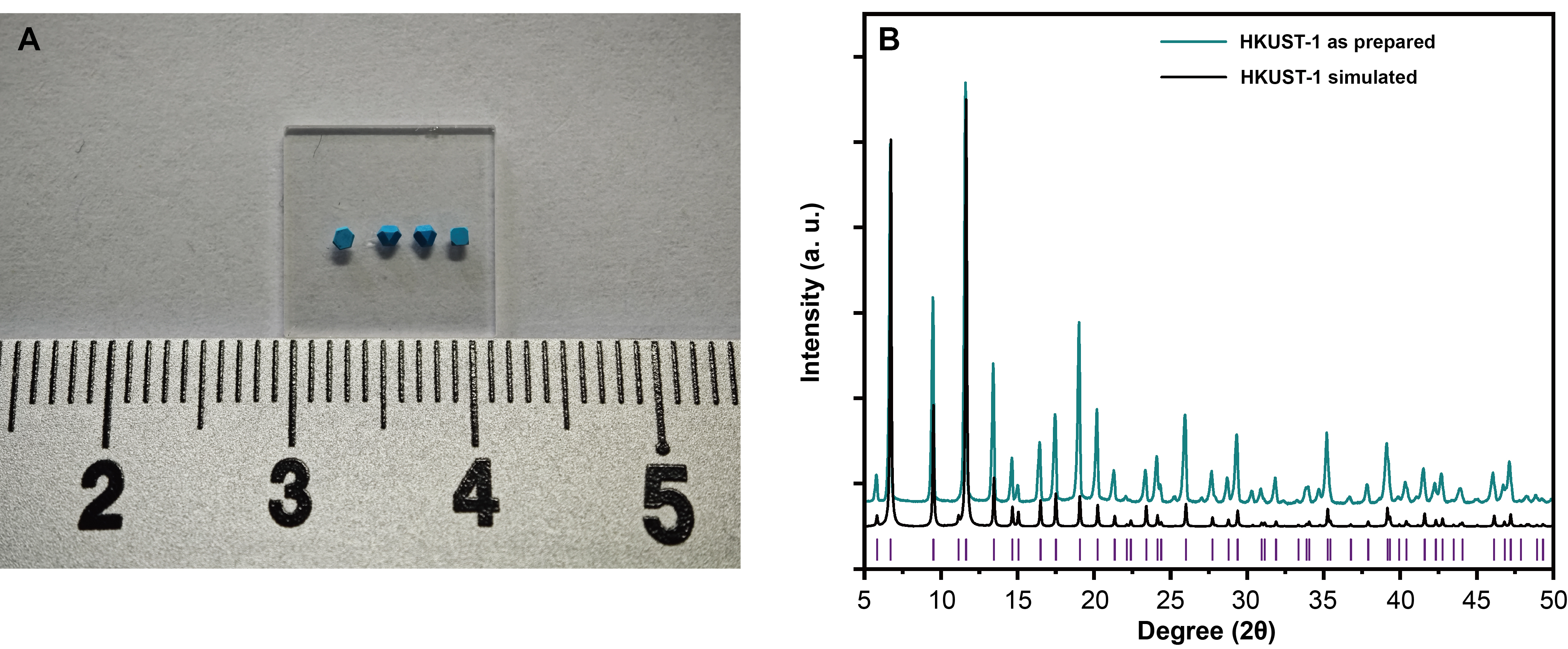
**

**Figure S1.** (A) Large HKUST-1 single crystal. (B) PXRD pattern of HKUST-1.

**
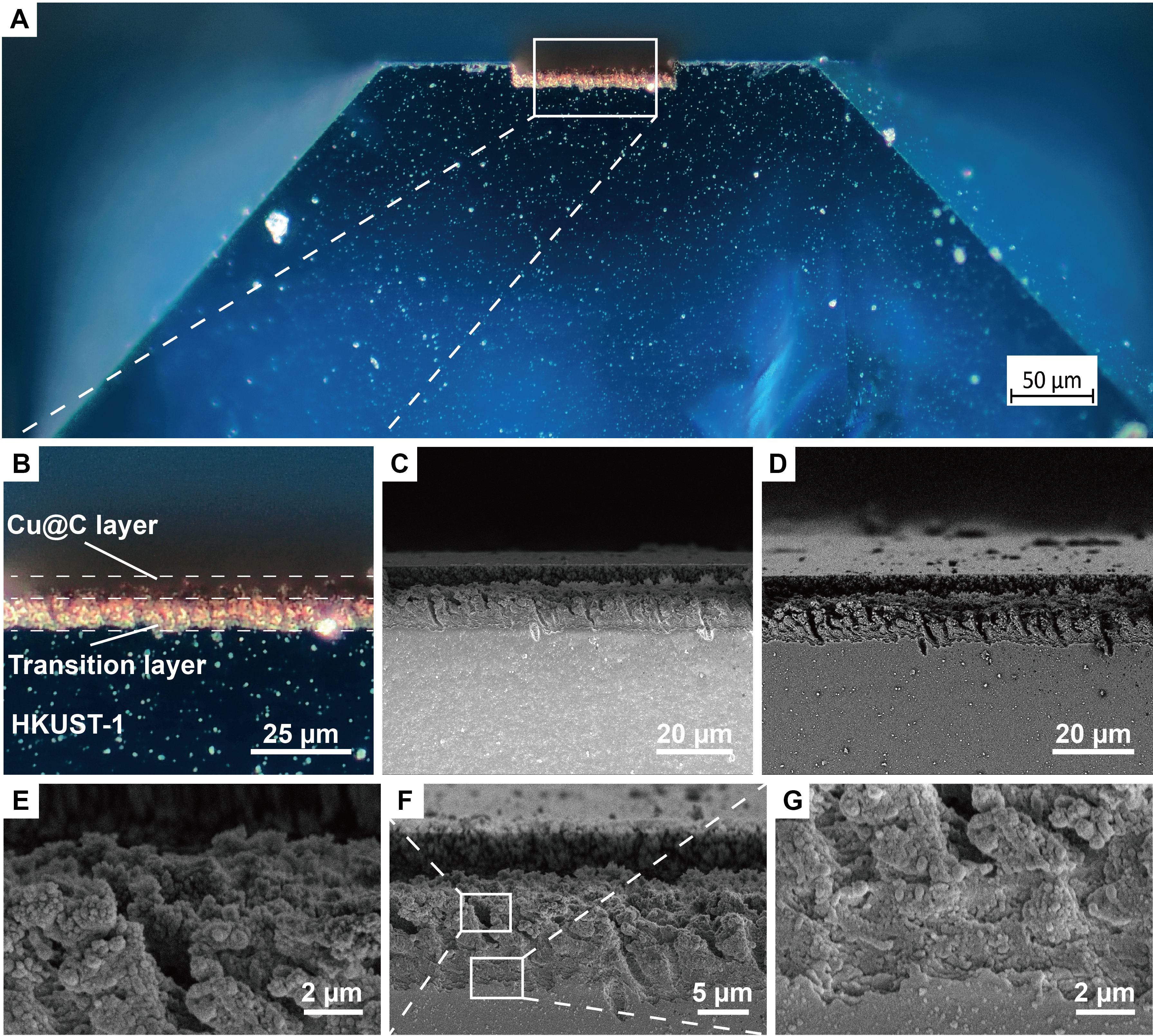
**

**Figure S2.** Side view images of the conductive interface. (A-B) Optical microscopy images. The reflected color gradually becomes lighter, indicating that the degree of metal reduction gradually decreases. SEM images using (C) secondary electron (SE) and (D) backscattered electron (BSE). (E-G) SEM images in SE, Cu@C NPs aggregate on the top of amorphous layer.


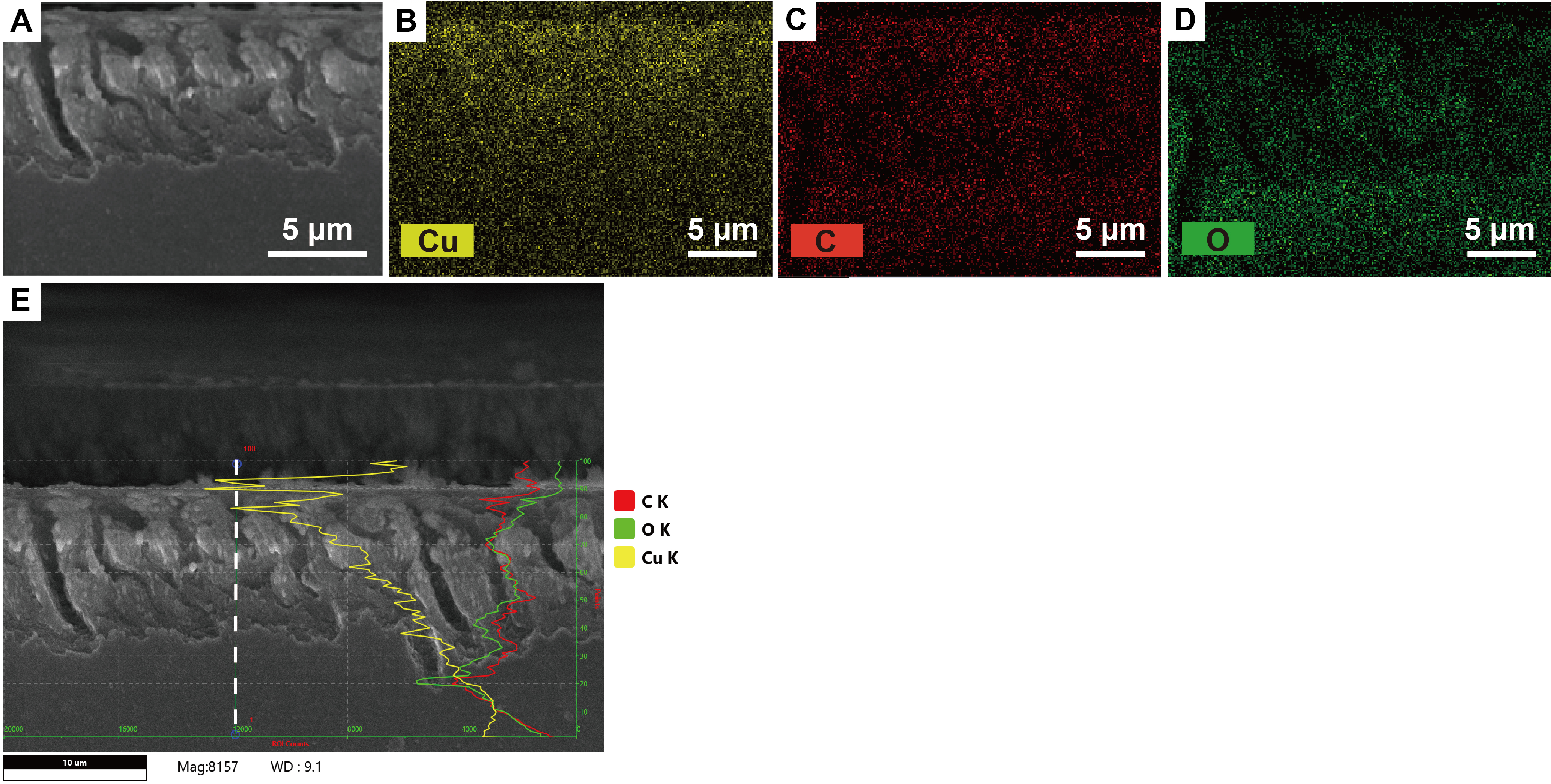


**Figure S3** EDS-SEM images of the conductive interface from the side view using (A-D) mapping and (E) line scan. The content of Cu decreases from the surface to the crystalline interface, which confirms the dissipation of reducing energy in depth.

**Table S1**. Representative fabrication strategies of MOF single crystal devices

| Method | Fabrication type | Precision | Procedure | Additive | Interface |
| --- | --- | --- | --- | --- | --- |
| **Ultrafast laser induced processing** | **Direct writing of patterning conductive structures** | **Microscale (3D single crystal)** |  |  | **In-situ continuous transition interface** |
| Two-photon 3D printing or photocatalysis | Additive manufacturing of polymer/metal inside crystal | Microscale (3D single crystal) | Pre-treated MOFs | Monomers, initiators or salts |  |
| X-ray and electron-beam lithography | Subtractive patterning | Microscale (3D single crystal) | Photomask and development |  |  |
| Electron beam or thermal evaporator | Metal electrodes patterning deposition | Nanoscale (2D single crystal) | Pre-patterned substrate, photomask and development | Photoresist | Electrode-crystal physical interface |
| Physical bonding | Direct metallic paste/electrode contact |  |  |  | Electrode-crystal physical interface |
| Transfer printing | Direct embedded structures via in-situ synthesis | Microscale (3D single crystal) | Pre-patterned substrate |  | Structure-crystal physical interface |

**
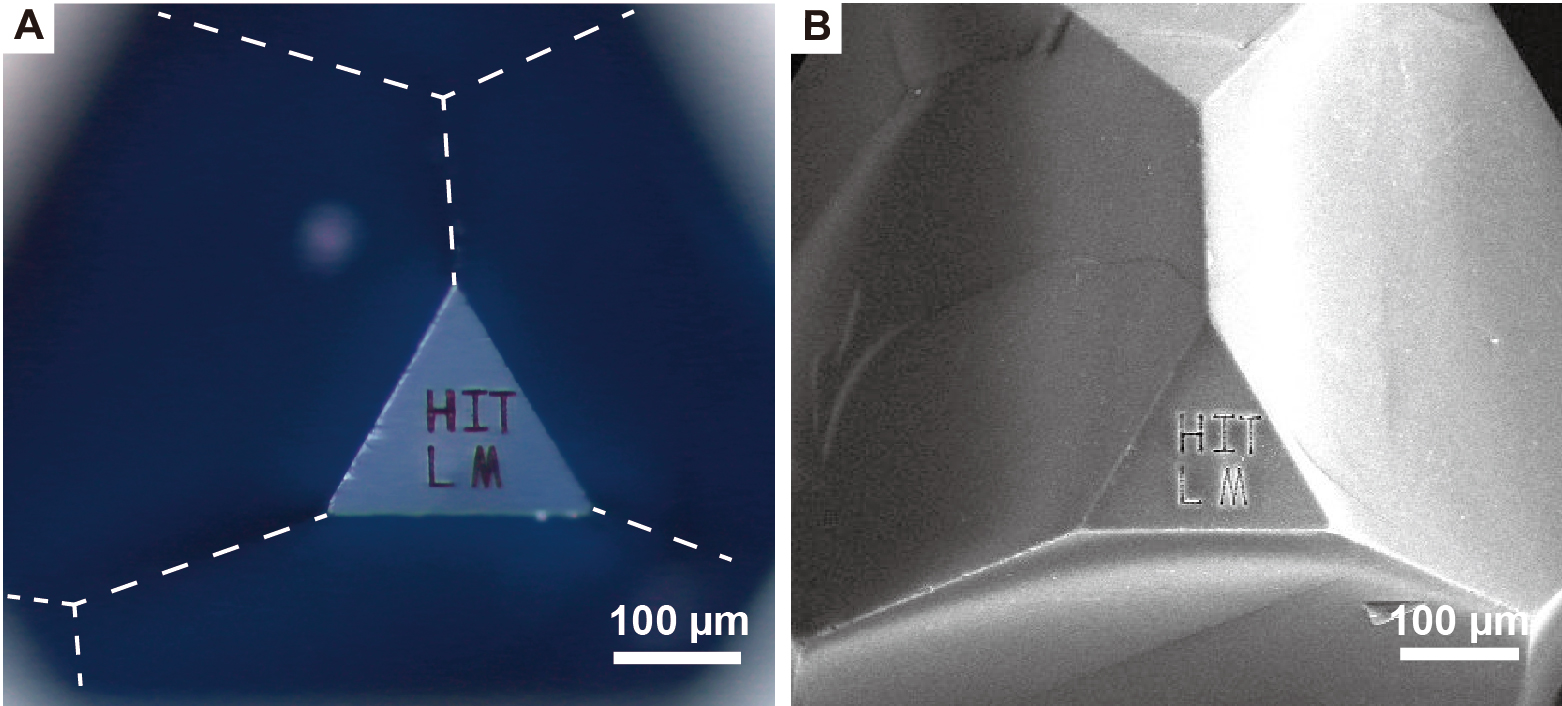
**

**Figure S4.** (A) Optical microscopic and (B) SEM images after 3 mW laser irradiation.

**
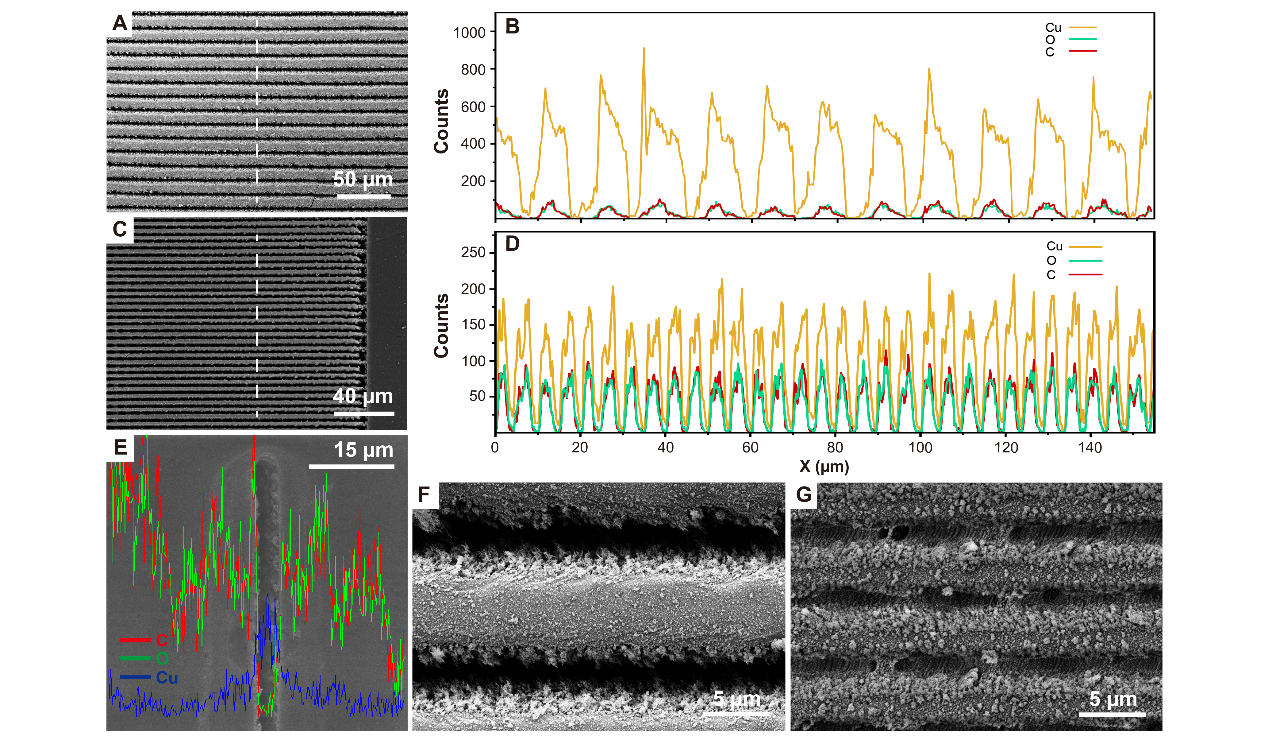
**

**Figure S5.** SEM images of (A) 9 mW laser processing with equal 13 μm interval and (C) 3 mW laser processing with 5 μm interval. Low magnification EDS-SEM line scan shows the degree of thermal reduction of Cu in the unprocessed spacer at (B) 9 mW and (D) 3 mW, while (E) high magnification line scan demonstrates Cu content within a single groove after 3 mW laser irradiation. High magnification SEM images after (F) 9 mW and (G) 3 mW processing.


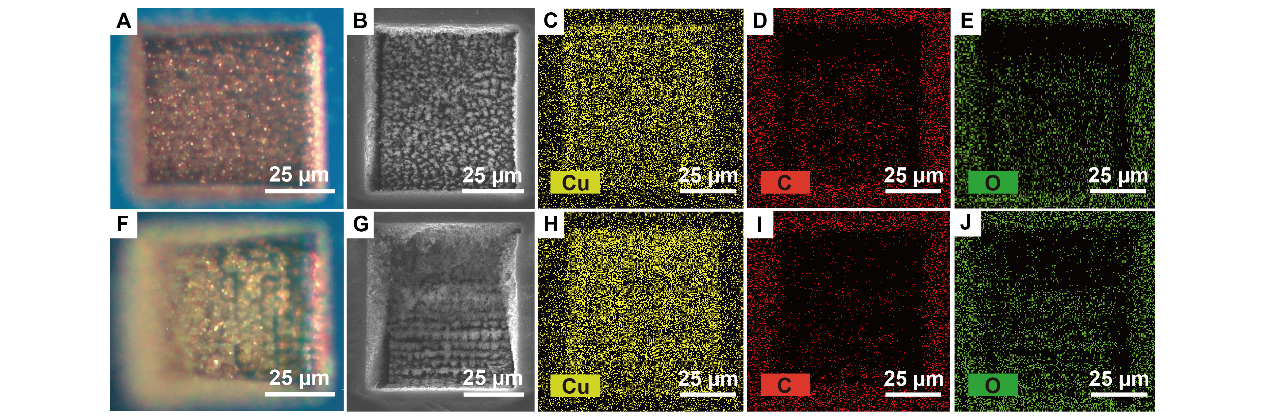


**Figure S6.** Optical microscope images and EDS-SEM images of 0.2 μm equal interval processing at (A-E) 3 mW and (F-J) 9 mW.

**
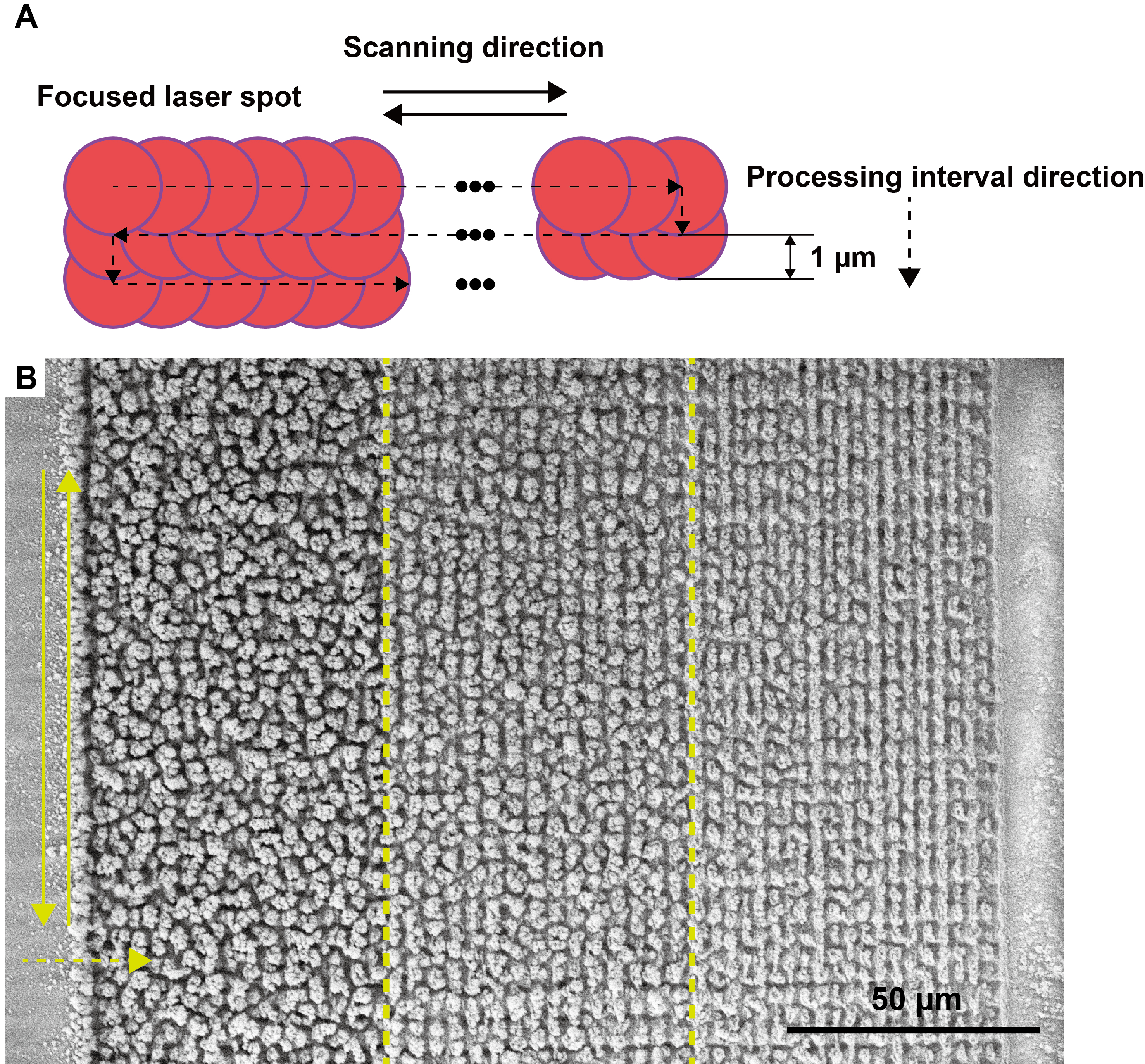
**

**Figure S7.** (A) The diagram of laser processing and (B) SEM image processed line-by-line at 3 mW laser power. The intervals from left to right are 1 μm, 2 μm and 3 μm, respectively. Smaller intervals are more conducive to a complete processing area, but a too small interval can lead a lower efficiency like 0.2 μm. Therefore, the interval chosen for the experiment is 1 μm.


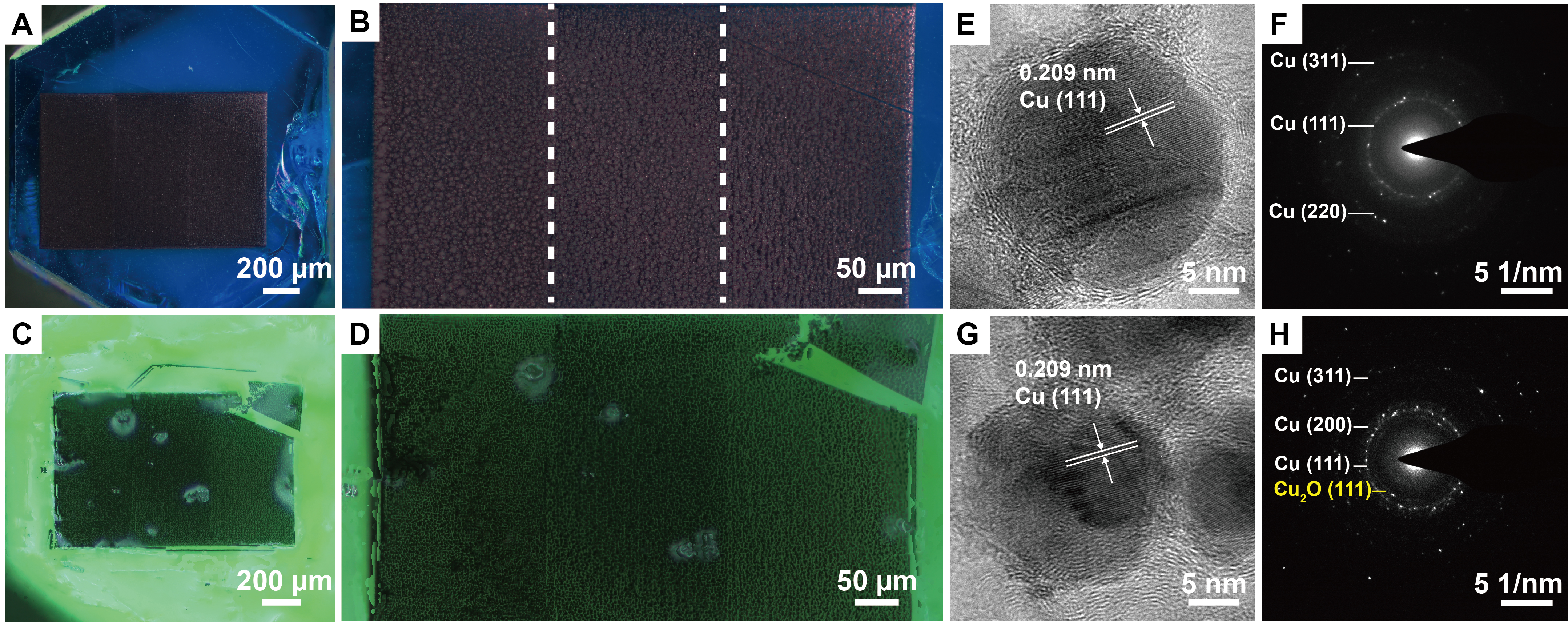


**Figure S8.** (A, B) Optical microscope image after laser irradiation. From left to right, the laser power is 3 mW, 5 mW and 8 mW, respectively. (C, D) Concentrated hydrochloric acid immersion. Colors of these regions are similar under the optical microscope and the morphology after immersion is similar, suggesting that the derived material is mainly Cu@C. HR-TEM images and SAED patterns at (E, F) 5 mW and (G, H) 8 mW reflect that with increasing heat accumulation, Cu_2_O nanoparticles begin to be produced.


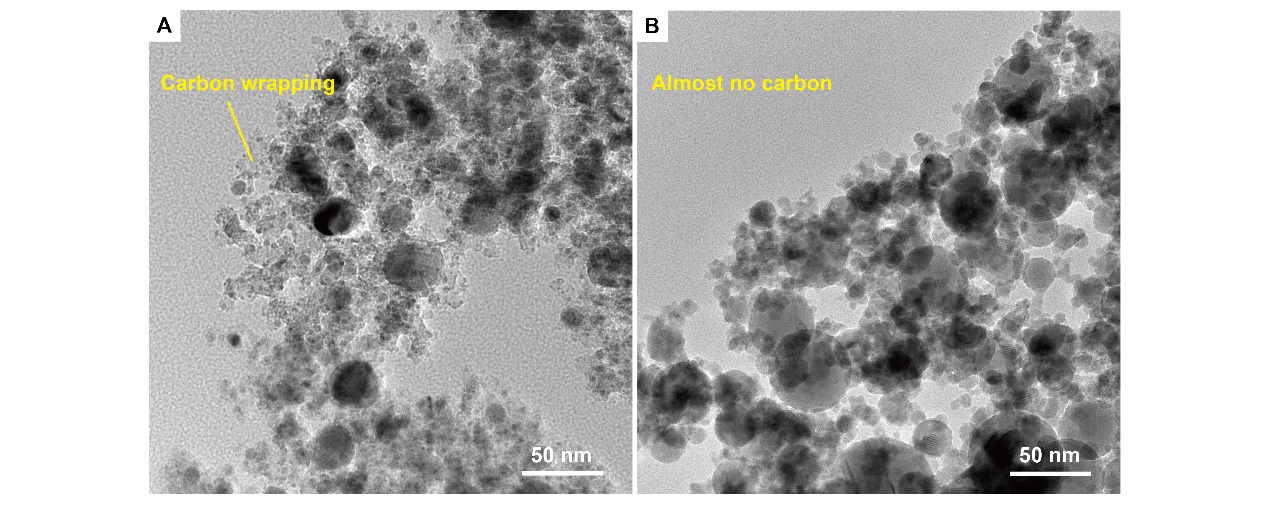


**Figure S9** TEM images at (A) 5 mW and (B) 9 mW laser power.


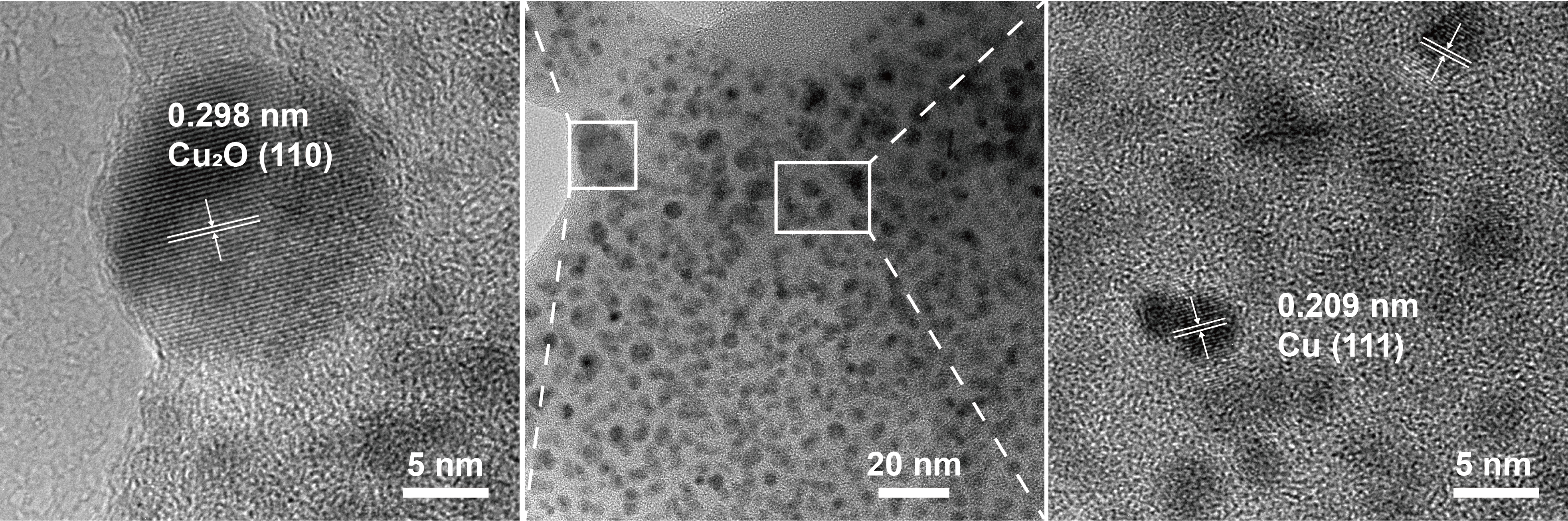


**Figure S10** HR-TEM images of 9 mW irradiated derivative material after submergence in ethanol and ultrasonic oscillation.


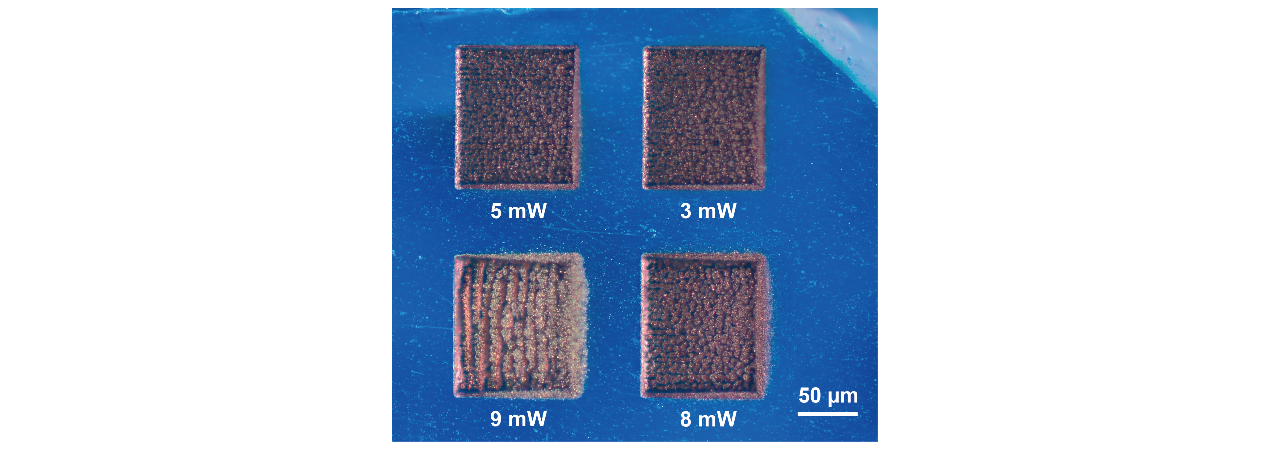


**Figure S11** Optical microscope image with different power conditions.


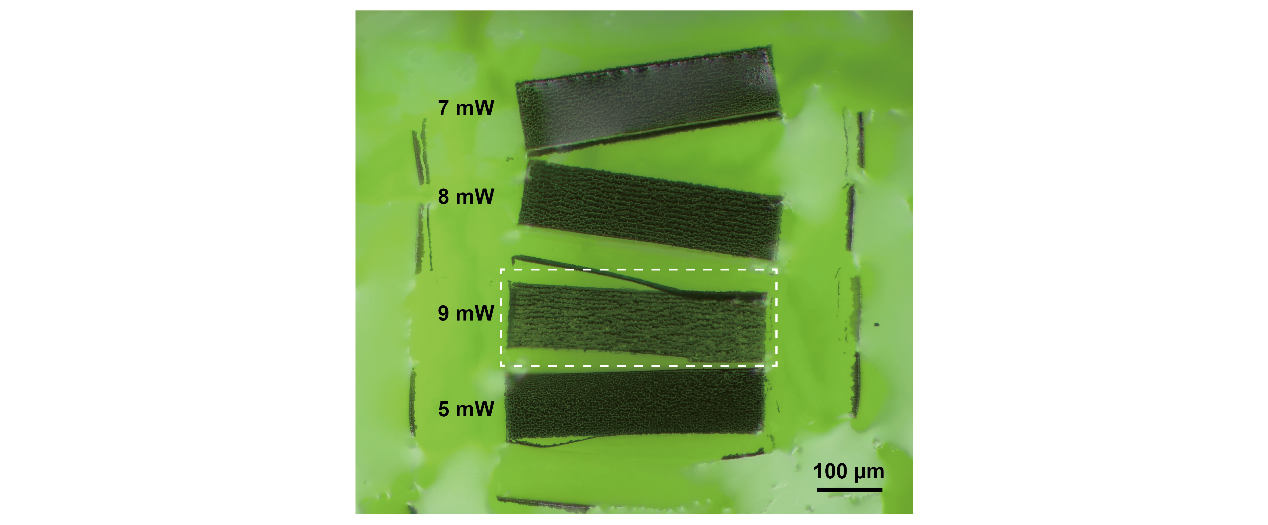


**Figure S12** Concentrated hydrochloric acid immersion for different processing powers. Cu_2_O produces earthy yellow complexes with concentrated acid while Cu@C can still be stabilized, clearly reflecting the degree of thermal oxidation of Cu NPs at 9 mW compared to other powers (dashed box).


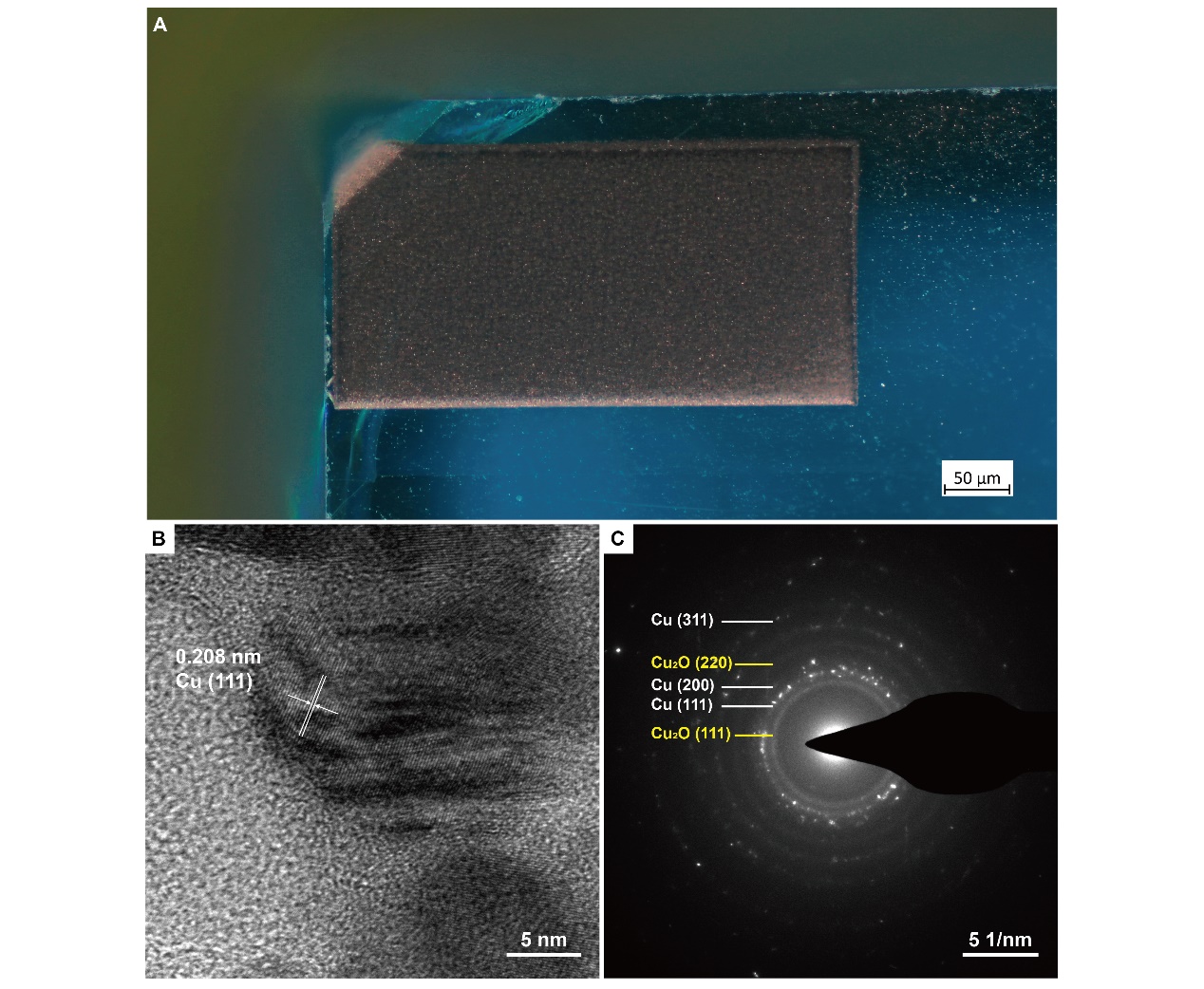


**Figure S13** (A) Optical microscope image and (B) HR-TEM, (C) SAED images at 3 mW, 20 μm s^-1^. By comparing the diffuse halos of the Cu_2_O phase with the apparent diffraction spots of the Cu phase in the SAED image and the color presented in the optical microscope, it can be concluded that the derived material produced under this condition is predominantly Cu and to a lesser extent Cu_2_O, in addition to carbon.

**
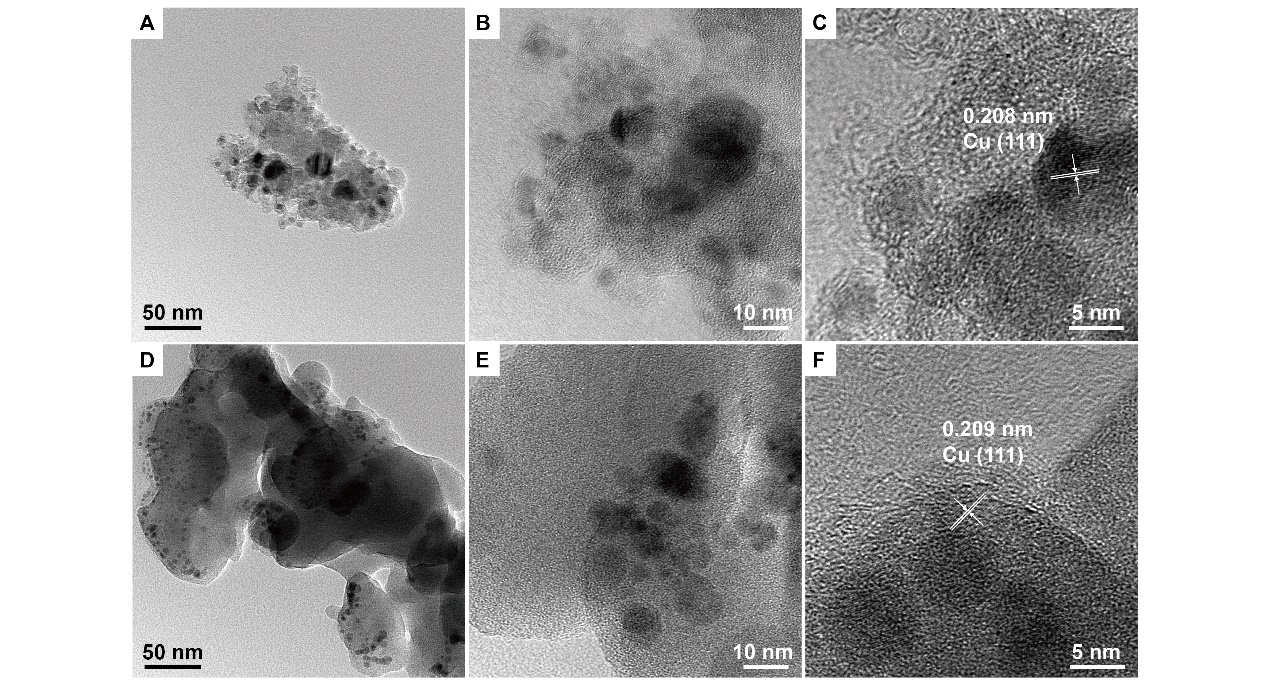
**

**Figure S14** SEM images of Cu@C obtained by single scan processing for (A-C) 3 mW and (D-F) 9 mW at 20 μm s^-1^, respectively. There is no obvious carbon ablation in a single scan and diffraction fringes of metallic Cu are still obtained, but there is a distinct lack of graphitization in the 9 mW-treated carbonaceous material compared to **Figure 2E-2H** (10 times reduction in heat accumulation).

**
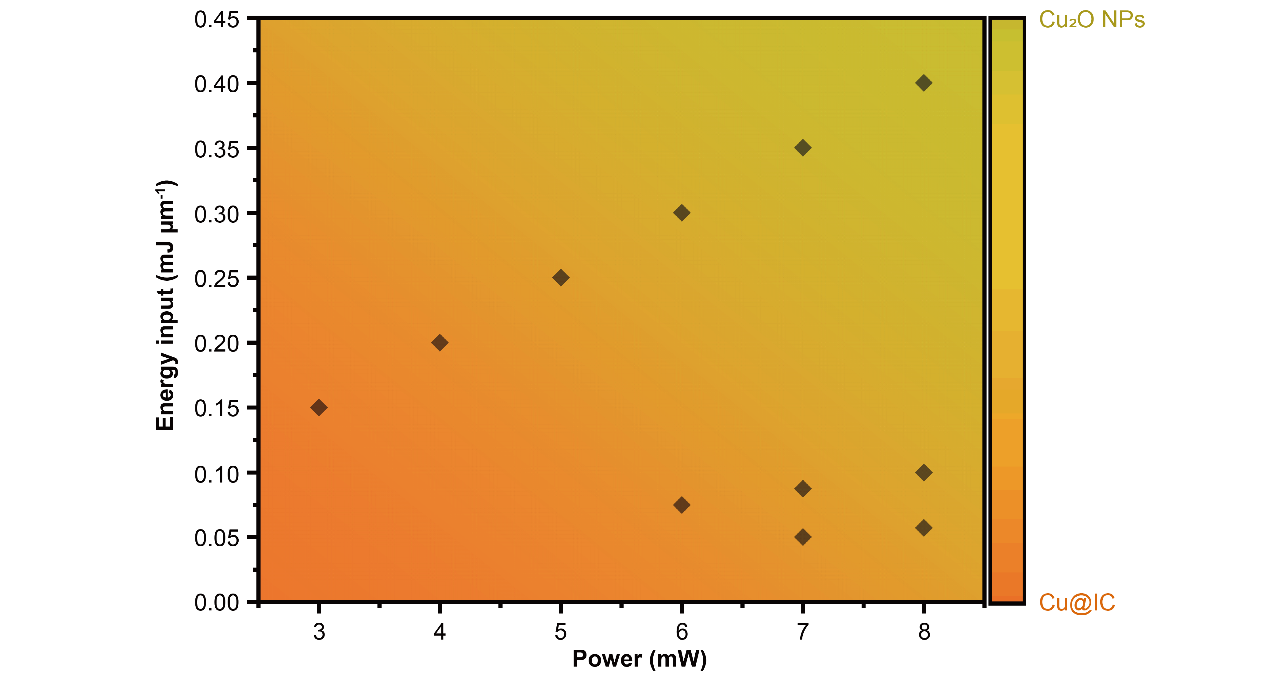
**

**Figure S15** Effect of heat accumulation on the transformation of Cu@Insulating Carbon to Cu_2_O.

**
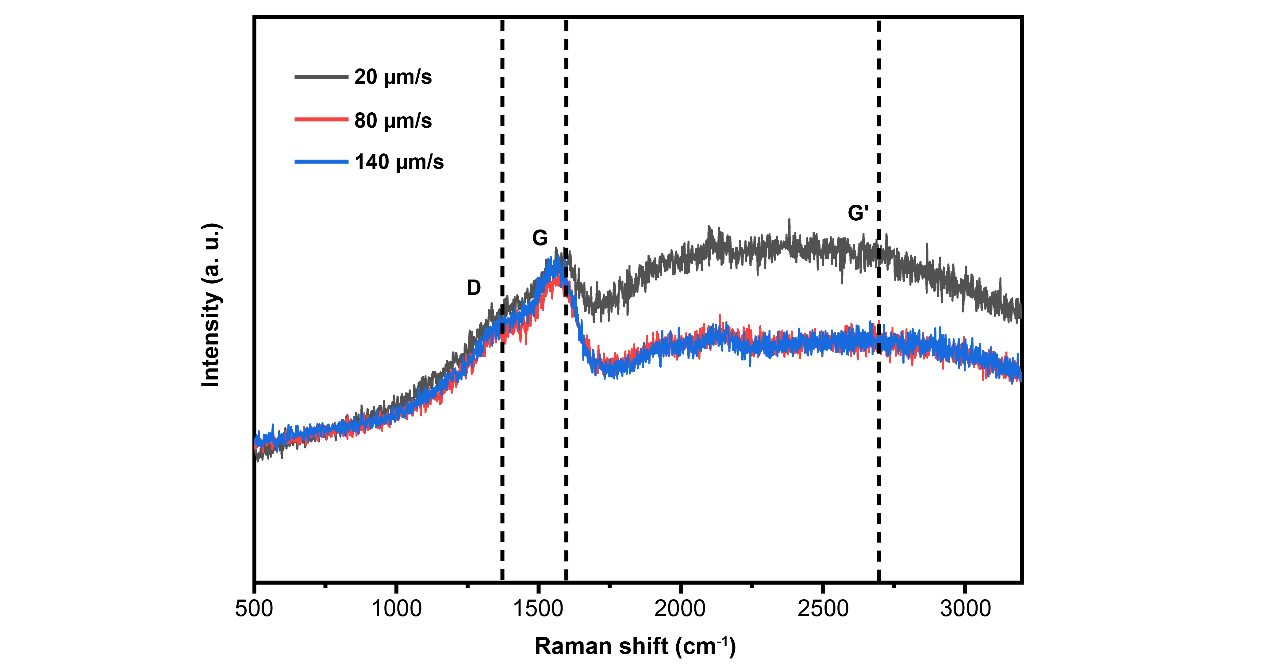
**

**Figure S16** Raman spectra after processing at 3 mW with different scanning speeds. The diffuse broadband (1700 cm^-1^~3000 cm^-1^) with intensity higher than that of the G band is observed in Cu@IC layer processed at 20 μm s^-1^, while the intensity of the corresponding broadband of Cu@C processed at 80 and 140 μm s^-1^ are significantly smaller than that of the G band. The current processed at 80 μm s^-1^ (current of 3.8 μA) and 140 μm s^-1^ (current of 98.7 μA) differ by a factor of at least 20, but the diffuse broadbands are similar due to the fact that they are all electrically conductive. However, the broadband of the insulating layer produced at 20 μm s^-1^ are significantly different from those of the conductive layer, which confirms the structural variability of the carbonaceous material in the insulating and conductive situations.^[4]^


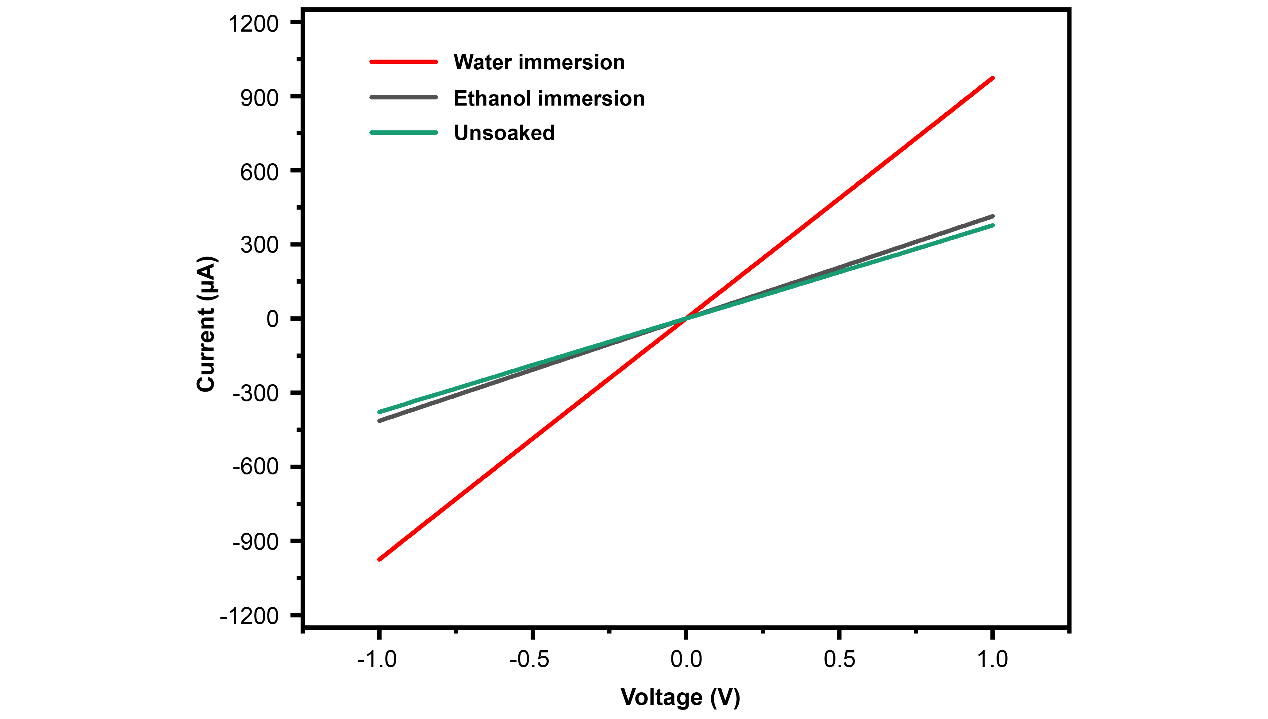


**Figure S17** I-V measurement of processing at 5 mW after direct immersion in water and ethanol, respectively. Direct immersion in water yields a current that is approximately three times the original current while immersion in ethanol produces no change in current.


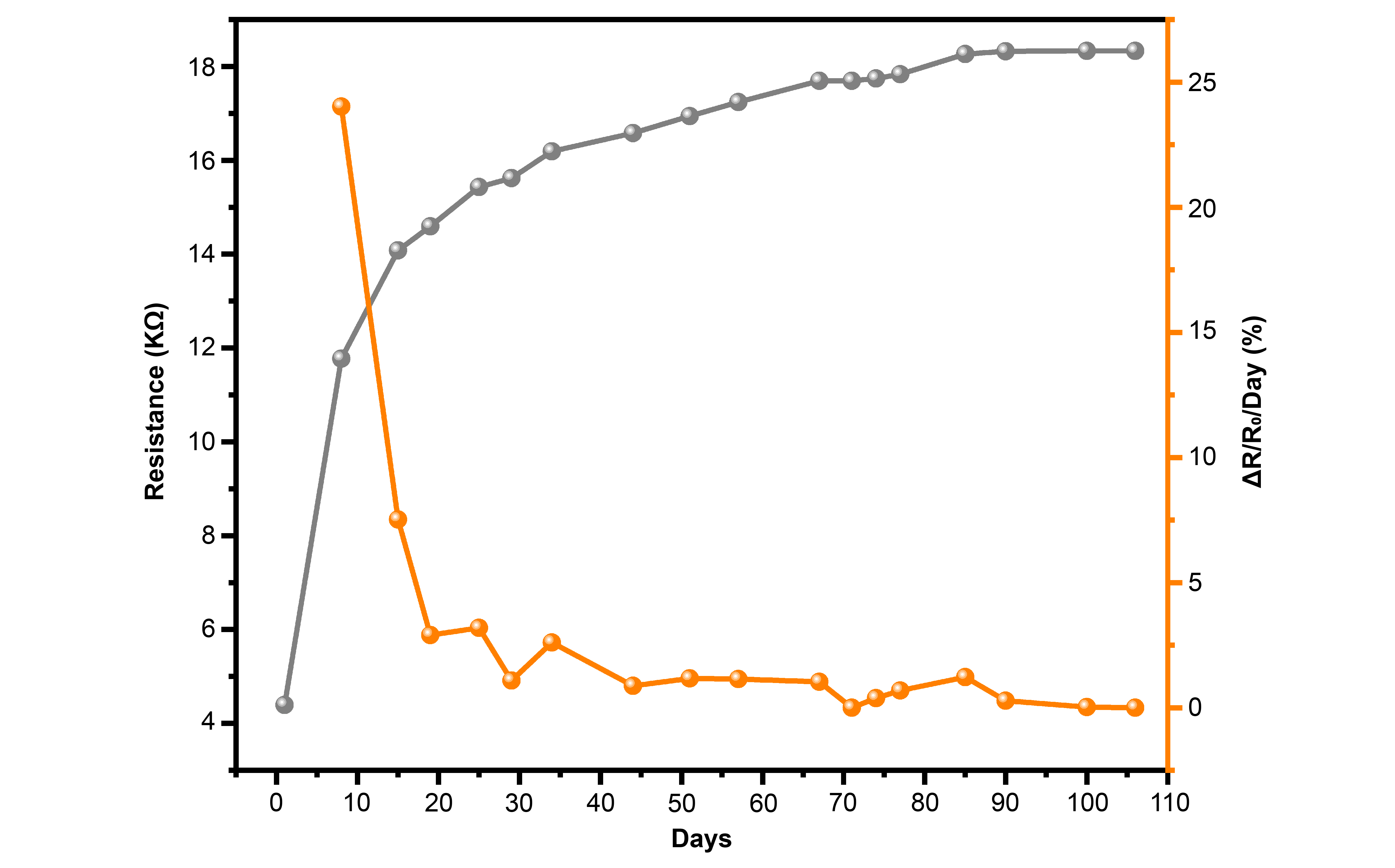


**Figure S18** Resistance value and growth rate of 3mW at 200 μm s^-1^ are tested. R_0_ is 4.39 KΩ (initial resistance), and the actual contact resistance between the copper wire and the Cu@C layer is consistent with the resistance performed in two-probe test. The test sample was left in air to oxidize naturally. It can be observed that the resistance value of the sample stabilizes around 18 KΩ, and the average oxidation rate gradually decreases.


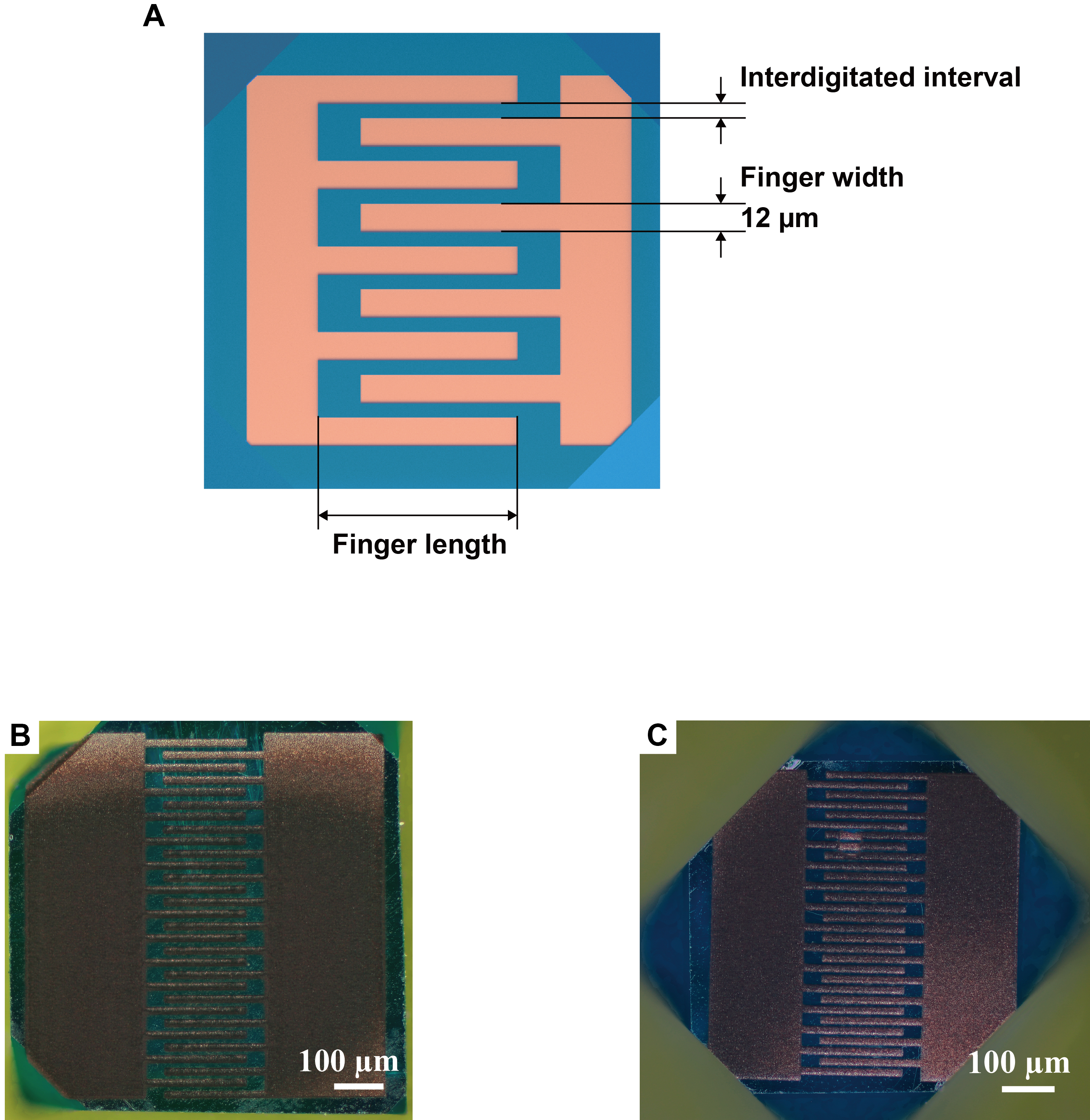


**Figure S19** (A) The diagram of electrode structures. A different interdigitated interval and aspect ratio may influence the sensing process. The aspect ratio is defined as the ratio of finger length to width. Optical microscopy images of IDEs on single crystals with different feature sizes: (B) 10 μm-50:3 and (C) 5 μm-50:3.


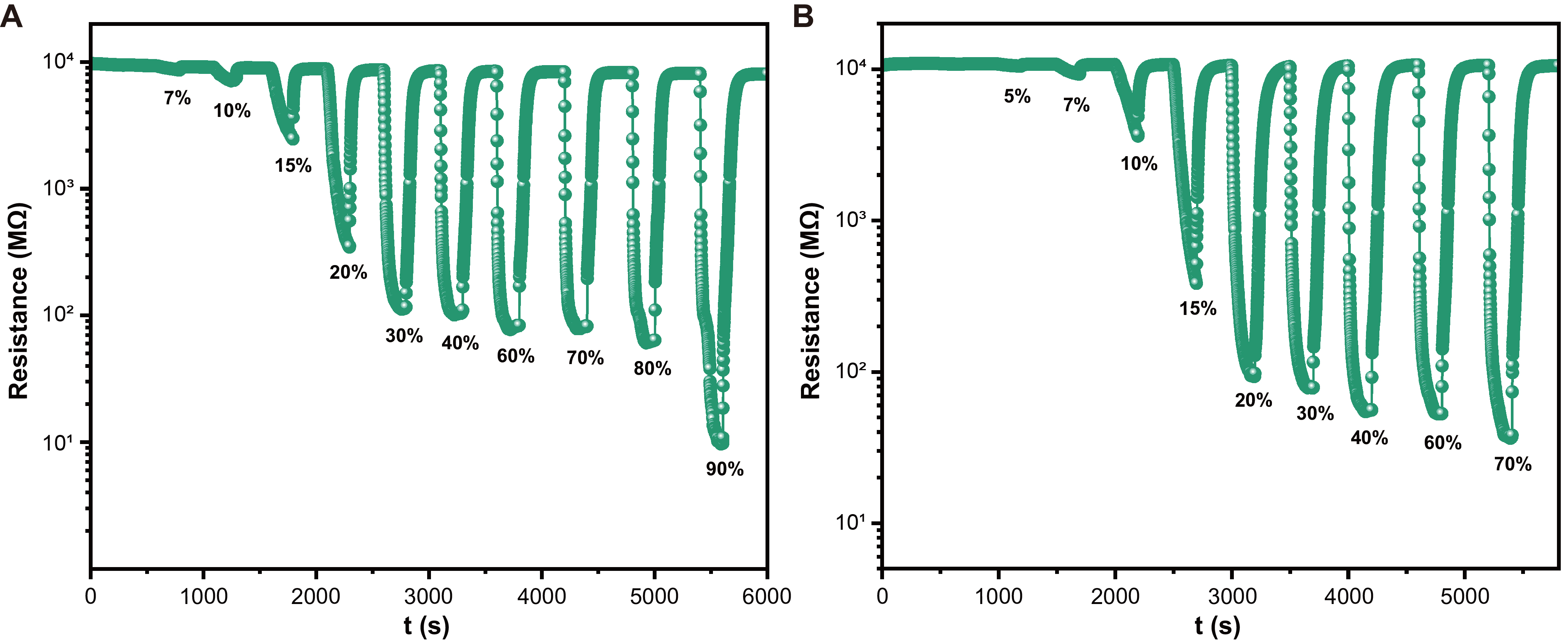


**Figure S20** Continuous response/recovery humidity test curves for different electrode configurations. (A) Interval of 5 μm, 75:3, (B) interval of 10 μm, 75:3, processed at 5 mW.

**Table S2.** Performance of HKUST-1 humidity sensors based on different material morphologies and fabrication strategies

| Type | Range | Sensitivity | Response time | Recovery time | Sample | Fabrication strategy | Ref |
| --- | --- | --- | --- | --- | --- | --- | --- |
| **Resistance** | **5%~90% RH** | **R_0_/R ~10^3^**  **(90% RH)** | **2~8 s**  **(30%~90%RH)** | **21~178 s** | **Single crystal** | **Laser direct writing micron IDEs on single HKUST-1** | **This work** |
| Dielectric constant |  | 2.95 ~ 57  (30% RH) | 160 s  (30% RH) | 395 s  (70%RH) | Single crystal | Direct probe contact | [5] |
| Capacitance | 11.3%~84.3% RH | 130.6 pF ~253.6 pF (84.3% RH) | 20 s | 20 s | Thin film | Growing on Cu slice | [6] |
| Capacitance | 20~100 ppm | 1.13 pF/ppm, ΔC/C_0_ ~0.5 (100 ppm) |  |  | Thin film | Electrochemical in-situ growth on Cu electrodes | [7] |
| Phase shift | 0.01%~42.5% RH |  | Hours | Hours | Thin film | LBL deposition on SAW devices | [8] |
| Deformation | 0.18%~0.98% mole |  | < 60 s | < 60 s | Thin film | Growing on microcantilevers | [9] |
| Absorbance |  |  | 23 s  (2.5 ppmv) |  | Film | Fiber optic gas sensor | [10] |
| Reflectance | 0%~10% RH |  | 300 s | 300 s | HKUST-1/polymer thin film | Spin-coating onto substrates | [11] |
| Frequency | 5%~75% RH | Δf/f_0_ ~2×10^-3^ | ~250 s | ~265 s | CNT-HKUST-1 thin film | Spin coating on QCM electrodes | [12] |
| Drain current | 0%~30% RH | I/I_0_ ~10  (30% RH) | 0.23 s  (30%RH) | 2.91 s | HKUST-1/P3HT thin film transistor | Evaporating electrodes onto films | [13] |
| Resistance | 11%~85% RH | R/R_0_ ~62 (85% RH) |  |  | HKUST-1/GO film | Deposition on Ag IDEs | [14] |
| Spectra shift | 0~12000 ppm |  |  |  | Silica colloidal crystal hybrid film | Deposition on glass slides | [15] |
| Impedance | 11%~95% RH | R/R_0_ ~100 (95%RH) | 2 s  (95% RH) | 15 s | Ultrathin nanosheet | Dip-coating on Ag IDEs | [16] |
| Impedance | 2%~20% RH | Z_0_/Z ~53.7 | 29 s  (20% RH) | 39 s | Au-HKUST-1 particles | Dropped onto graphite IDEs | [17] |
| Frequency | 22%~69% RH | Δf (Hz) ~720 | 1676 s | 1051 s | Particles | Drop casting on QCM electrodes | [18] |


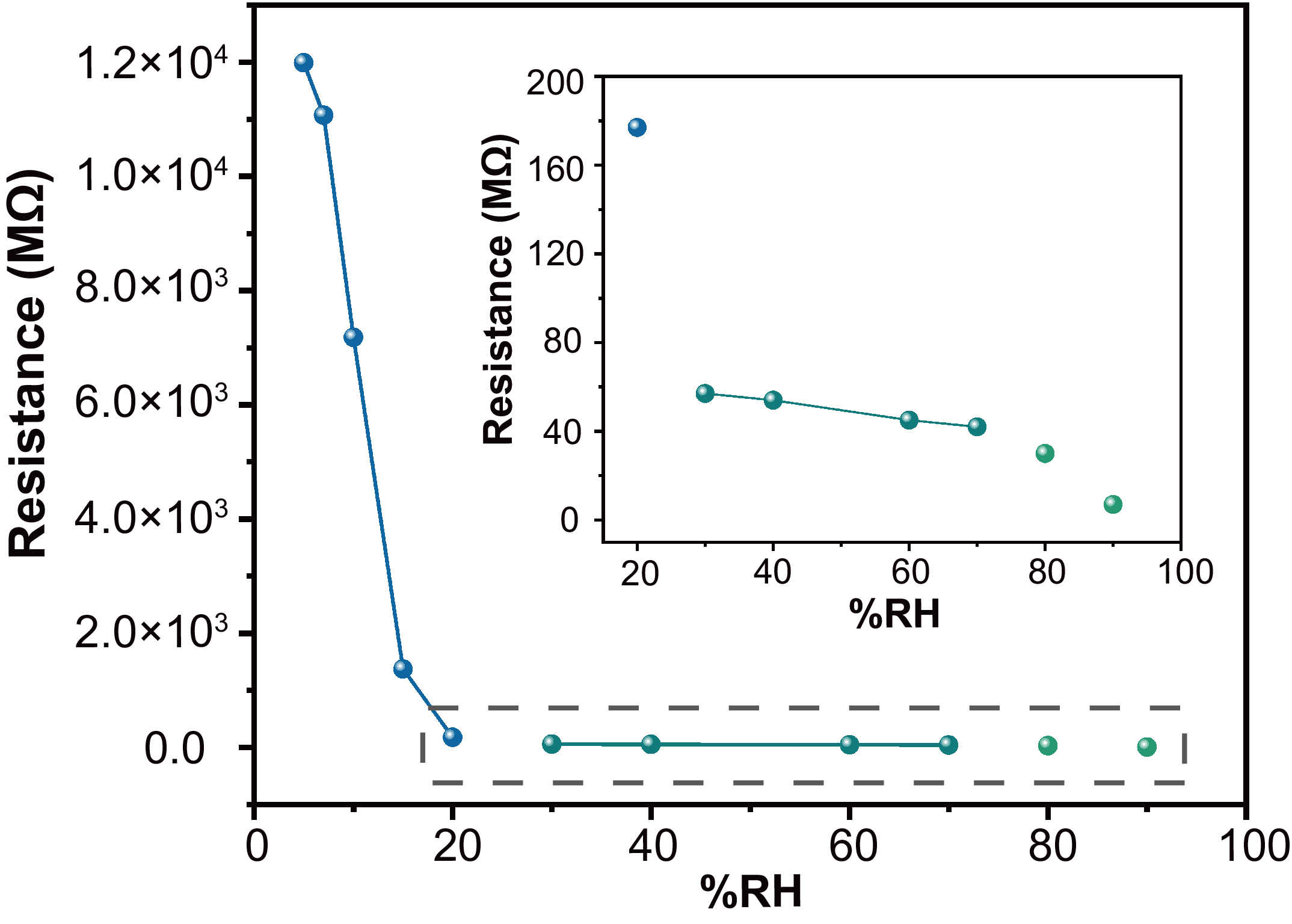


**Figure S21** Response-humidity curves.

**References**

[1] G. Kresse, J. Furthmüller, *Phys. Rev. B Condens. Matter* **1996**, *54*, 11169.

[2] J. P. Perdew, K. Burke, M. Ernzerhof, *Phys. Rev. Lett.* **1996**, *77*, 3865.

[3] G. Kresse, D. Joubert, *Phy. Rev. B* **1999**, *59*, 1758.

[4] L. M. Malard, M. A. Pimenta, G. Dresselhaus, M. S. Dresselhaus, *Phy. Rep.* **2009**, *473*, 51.

[5] A. S. Babal, A. K. Chaudhari, H. H.-M. Yeung, J.-C. Tan, *Adv. Mater. Interfaces* **2020**, *7*, 2070080.

[6] J. Liu, F. Sun, F. Zhang, Z. Wang, R. Zhang, C. Wang, S. Qiu, *J. Mater. Chem.* **2011**, *21*, 3775.

[7] M. S. Hosseini, S. Zeinali, *J. Mater. Sci. Mater.* **2019**, *30*, 3701.

[8] A. L. Robinson, V. Stavila, T. R. Zeitler, M. I. White, S. M. Thornberg, J. A. Greathouse, M. D. Allendorf, *Anal. Chem.* **2012**, *84*, 7043.

[9] M. D. Allendorf, R. J. T. Houk, L. Andruszkiewicz, A. A. Talin, J. Pikarsky, A. Choudhury, K. A. Gall, P. J. Hesketh, *J. Am. Chem. Soc.* **2008**, *130*, 14404.

[10] S.-I. Ohira, Y. Miki, T. Matsuzaki, N. Nakamura, Y.-k. Sato, Y. Hirose, K. Toda, *Anal. Chim. Acta.* **2015**, *886*, 188.

[11] A. M. Ullman, C. G. Jones, F. P. Doty, V. Stavila, A. A. Talin, M. D. Allendorf, *ACS Appl. Mater. Interfaces* **2018**, *10*, 24201.

[12] K. N. Chappanda, O. Shekhah, O. Yassine, S. P. Patole, M. Eddaoudi, K. N. Salama, *Sensor. Actuat. B-Chem.* **2018**, *257*, 609.

[13] Y. J. Jang, Y. E. Jung, G. W. Kim, C. Y. Lee, Y. D. Park, *RSC Adv.* **2019**, *9*, 529.

[14] W. Zhang, S. Meng, H. Wang, Y. He, *J. Phys. Conf. Ser.* **2018**, *986*, 012013.

[15] G. Lu, O. K. Farha, L. E. Kreno, P. M. Schoenecker, K. S. Walton, R. P. Van Duyne, J. T. Hupp, *Adv. Mater.* **2011**, *23*, 4449.

[16] Q. Wang, M. Lian, X. Zhu, X. Chen, *RSC Adv.* **2021**, *11*, 192.

[17] X. Miao, K. Wu, Y. Yu, Z. Ma, Y. Song, Y. Cui, S. Liu, T. Fei, T. Zhang, *IEEE Sens. J.* **2024**, *24*, 7497.

[18] L. Kosuru, A. Bouchaala, N. Jaber, M. I. Younis, *J. Sens.* **2016**, *2016*, 4902790.
